# 50 Years of Giese Reaction – a Personal View

**DOI:** 10.1002/anie.202524825

**Published:** 2025-12-12

**Authors:** Martin Spichty, Hendrik Zipse, Salem Majouri, Katharina M. Fromm, Bernd Giese

**Affiliations:** ^1^ Laboratoire d'Innovation Moléculaire et Applications (UMR 7042) Université de Strasbourg|Université de Haute‐Alsace|CNRS—IRJBD 3 bis rue Alfred Werner Mulhouse 68057 CEDEX France; ^2^ Department Chemie LMU Muenchen Butenandtstrasse 5–13 D‐81377 Muenchen Germany; ^3^ Department of Chemistry University of Fribourg Chemin du Musée 9 Fribourg 1700 Switzerland

**Keywords:** Cellular redox reaction, Giese reaction, Radical reaction, Reaction mechanism, Synthesis design

## Abstract

50 years ago, a synthetic method was discovered, in which alkyl radical precursors, alkenes and hydrogen donors selectively yield 1:1:1‐addition products in cyclic chain reactions. This paved the way for many variants of three‐component syntheses, which became standard procedures for C,C‐bond formation. For successful syntheses the different chain carrying radicals have to follow reactivity and selectivity rules. This requires knowledge of the substituent influence on substrate‐, regio‐ and stereoselectivities of intermolecular radical reactions. These rules were experimentally elucidated, and the synthetic method was coined “Giese reaction”. 20 years after its discovery in the chemical laboratory, biologists observed that microorganisms use the same synthetic strategy, which triggered our studies on biological cells. Although chemical rules in laboratory vessels and biological cells are the same, their different set‐ups lead to very different features. Syntheses in homogeneous solution of a laboratory vessel is driven by kinetic effects. In contrast, most reactions in biological cells occur at protein/water interfaces, where thermodynamic interactions with enzymatic amino acids establish close contact between the educts. In addition, biochemical processes often start with metallo‐cofactors that generate the productive radicals at the interface by long‐distance electron transfer.

## Graphical abstract

The Giese‐Reaction celebrates its 50. Birthday, it is a cyclic radical chain reaction centered around C,C‐bond formation in a three component synthesis. This minireview speaks about the birth of the name‐reaction, explains how the complete understanding of its reaction mechanism stimulates new methods in organic synthesis, and why it can proceed in living cells.

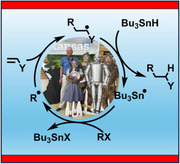

## 1 Introduction

**Scheme 1:**
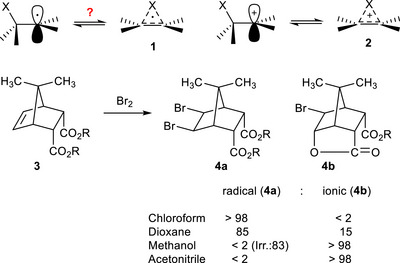
The question is raised whether ß‐substituted radicals can exist as bridged (non‐classical) radicals **1**. Bromination of norbornene **3** yields radical product **4a** and ionic product **4b**. In the absence of methyl groups at C‐7 only the bromolactone is formed. A methyl group at C‐7 shields the center of the double bond yielding dibromide **4a** in solvents of low polarity.

In 1972, Kaplan^[^ ^1^ ^]^ published a monograph, in which the question was raised, whether alkyl radicals with a ß‐C,X bond are open or bridged species **1** (Scheme 1). Spectroscopic and chemical experiments gave some evidences that bridged radicals might exist for X═Ge and Sn. At the same time Kochi^[^ ^2^ ^]^ and Symmons^[^ ^3^ ^]^ deduced from ESR data that also ß‐halogen atoms interact with the singly occupied orbital. During the habilitation work in the early 1970s I (B. G.) studied the probability of bridged (non‐classical) radicals **1** in comparison to bridged (non‐classical) cations **2**.

A simple experiment indicated the different importance of bridging for ionic and radical additions to alkenes (Scheme 1).^[^ ^4^ ^]^ Without the methyl groups at C‐7 of norbornene **3,** where a central attack at the double bond is possible, bromination yields only the ionic product. In contrast, the shielding methyl group in **3** shifted the reaction toward a radical pathway. To learn more about the influence of a ß‐C,X bond on the bridging effect in radicals, we searched for a more flexible radical generation. Whitesides^[^ ^5^ ^]^ suggested the reduction of organomercury salts, and we decided to synthesize ß‐substituted organomercury salts as precursors for ß‐substituted radicals. First, we tested whether reduction of organomercury salt yields intermediates that show the same features as radicals generated from traditional precursors. Two tests where selected: selectivity measurements of radical halogen abstraction, and radical initiated polymerization of alkenes. Both studies led to unexpected results.

## 2 Enthalpy‐Entropy Compensation

**Figure 1:**
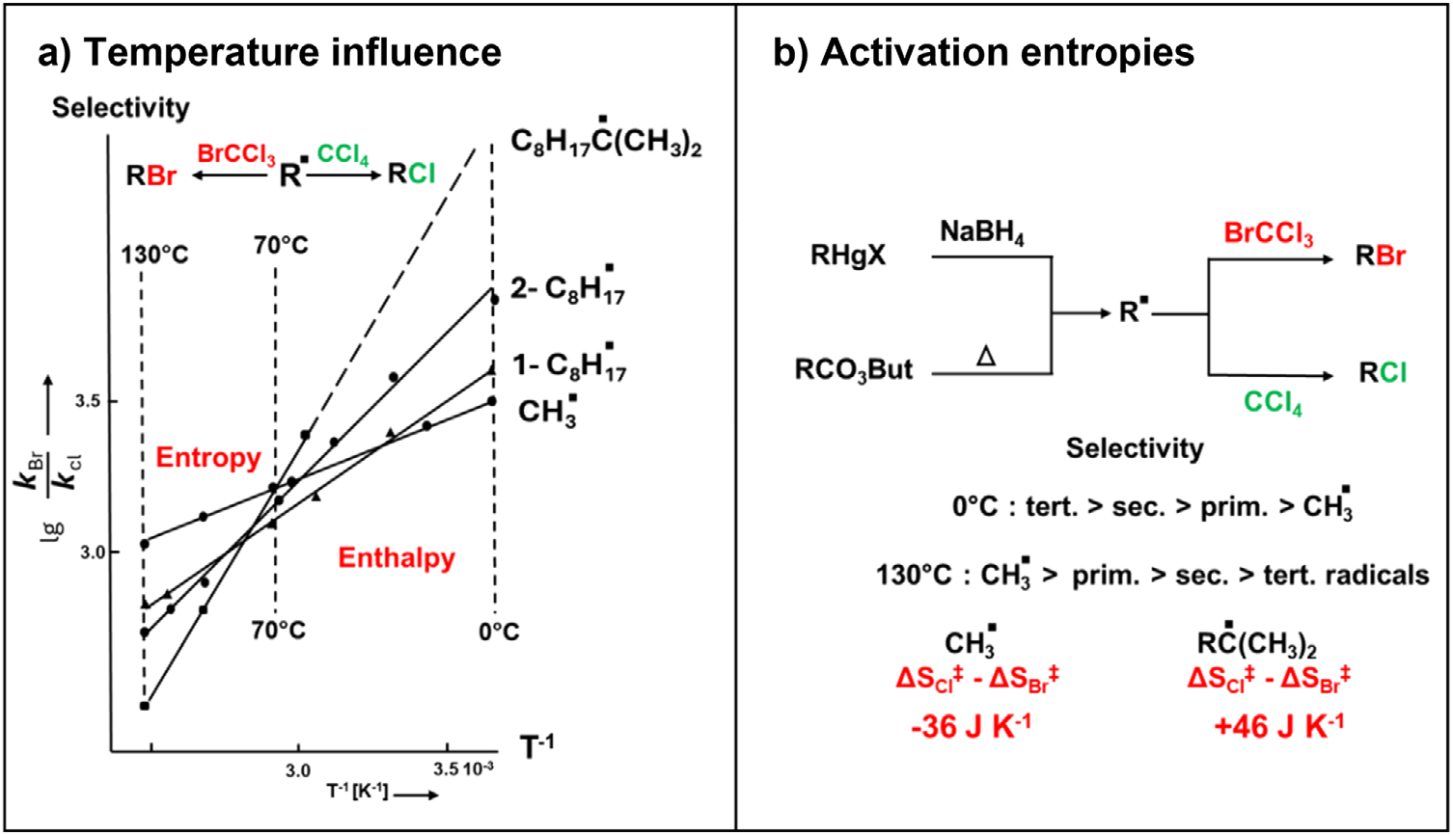
a) Temperature influence on alkyl radical selectivities, b) change of the selectivity sequence and differences in the activation entropies.

Reduction of alkylmercury salts RHgX in CCl_4_, containing small amounts of BrCCl_3_, yields RBr and RCl. To compare the data with those from radicals formed from peresters, temperature effects were measured (Figure 1a). Selectivity data between 0 °C (mercury method) and 130 °C (perester method) of each intermediate lay on the same straight line. This demonstrated that reduction of alkylmercury salts generates alkyl radicals as intermediates. The surprise however was, that the lines of the different radicals cross each other at 60 ± 20 °C.^[^ ^6^ ^]^ As a result, radical selectivities at 0 °C increase from methyl radical to the tertiary radical, but at 130 °C the selectivity order is turned around, and now the methyl radical is the most selective alkyl radical. In the small temperature region of 60 ± 20 °C, radical selectivities coincide and cannot be differentiated. The reason for this observation is a compensation of enthalpy effects by entropy effects. Enthalpy/entropy compensation is an often observed or predicted event in rate and equilibrium experiments,^[^ ^7^ ^]^ but our observation was exceptional as the reversal of the selectivity sequence occurs at a reaction temperature, at which these experiments were typically carried out. We called this correlation an “isoselective relationship” and the temperature of selectivity collapse the “isoselective temperature”.^[^ ^6^, ^8^ ^]^ The reason for the low isoselective temperature is a large variation of activation entropies of the different radicals. Figure 1b shows that in going from the methyl radical to a tertiary radical, the differences of activation entropies vary by 82 J K*^−1^* mol^−1^. This change is much too large to be explained by differences of conformational freedoms.

**Figure 2:**
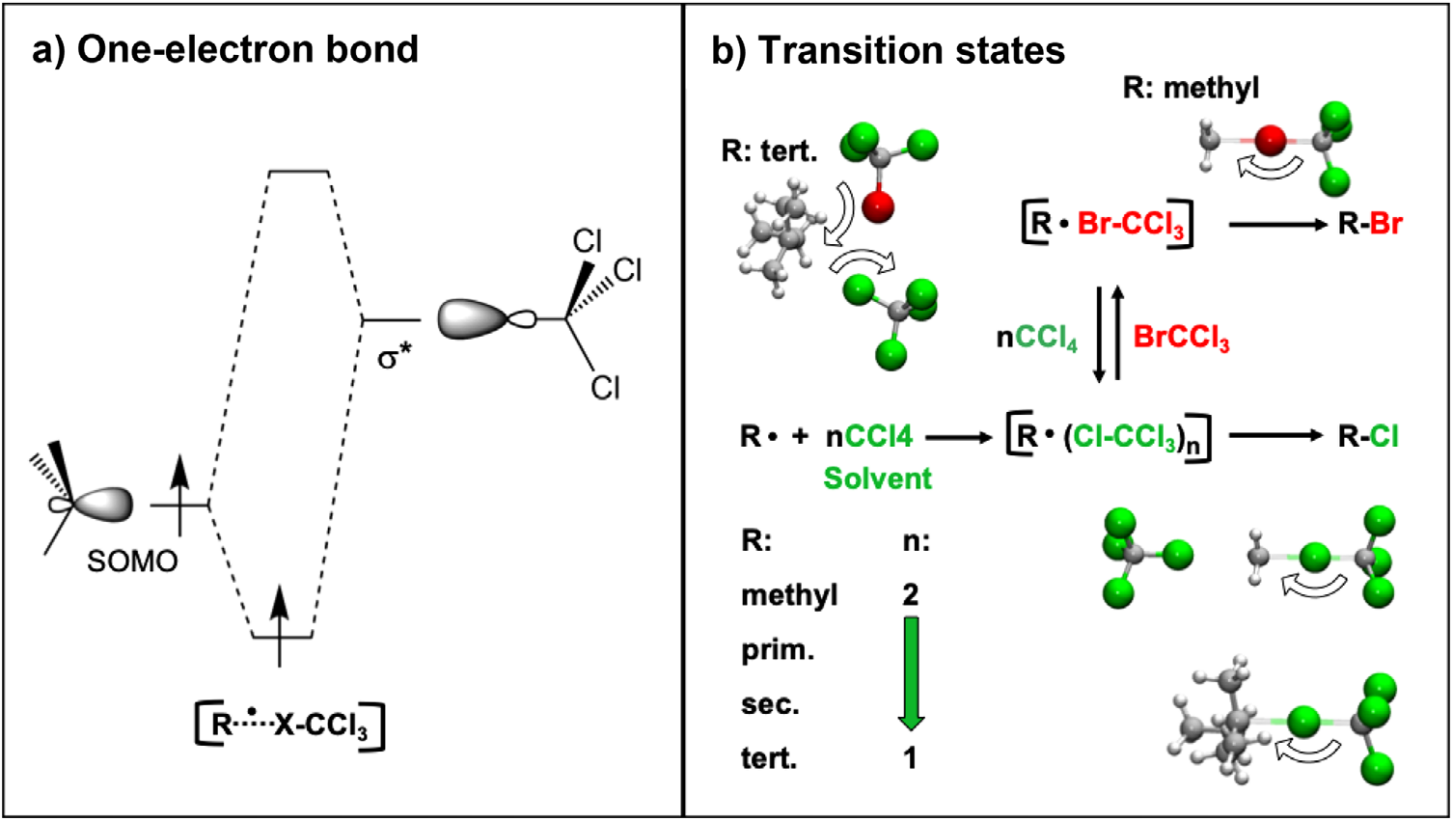
a) The SOMO interaction of a nucleophilic alkyl radical with the anti‐bonding σ*‐orbital of the XCCl_3_ halogen bond leads to a stabilizing one‐electron bond. b) Bottom: depending on its steric hindrance, the radical can be complexed by one or two CCl_4_ solvent molecules in the transition states of RCl formation. Two CCl_4_ molecules are bound by the small methyl radical. Top: transition states for the Br‐abstraction gradually shift with increasing rate from bimolecular (methyl radical) to trimolecular of the tert. radical for which the reaction is nearly diffusion controlled so that the addition of BrCCl_3_ to the complex is rate determining.^[^ ^9^ ^].^

We have now found a possible explanation^[^ ^9^ ^]^ which is based on the existence of intermediates that are formed by one‐electron bonds between alkyl radicals and halogen donors (Figure 2a).^[^ ^10^ ^]^ These “hemibond” interactions^[^ ^11^ ^]^ are weaker with chlorine (CCl_4_) than with bromine (BrCCl_3_). In the gas phase, the experimental findings are very different,^[^ ^12^ ^]^ they can be explained by conventional bimolecular transition states between halogen donors and radicals.^[^ ^9^ ^]^ But if experiments in CCl_4_‐solution lead to complex formation, the reactivity and steric effects of the radicals can decide on the number of additional solvent molecules in the rate‐determining transition states (Figure 2b), which obviously strongly impact the activation entropy.^[^ ^9^ ^]^ Because an entropy loss of complex formation is compensated by an enthalpy gain due to complex bond formation,^[^ ^13^, ^14^, ^15^, ^16^, ^17^ ^]^ the different number of complexed molecules in the relevant transition state of halogen abstraction leads to the enthalpy/entropy compensation. The details can be read in our work on DFT, MP2 and coupled‐cluster calculations.^[^ ^9^ ^]^

## 3 Giese Reaction

**Scheme 2:**
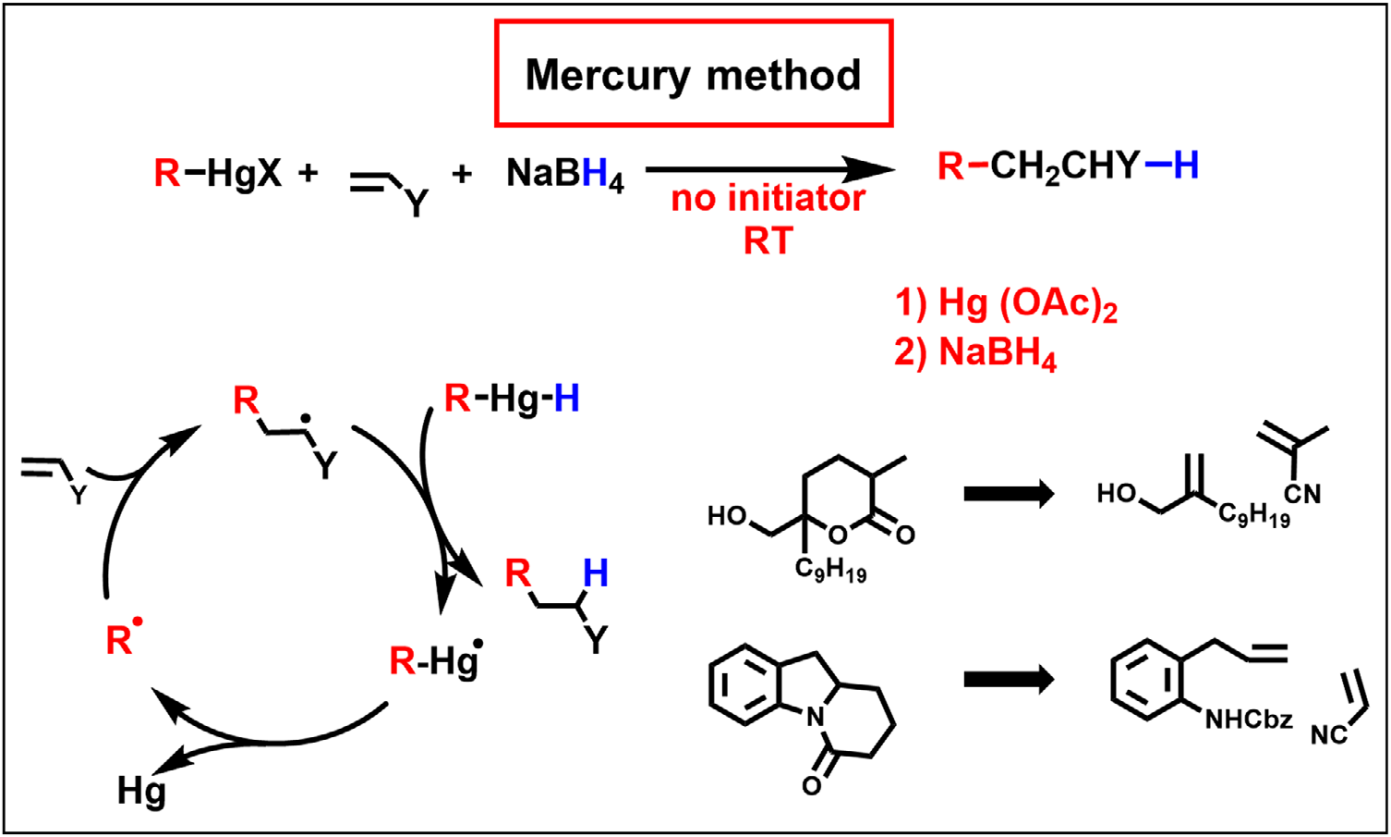
Giese reactions are cyclic chain reactions between radicals, alkenes and hydrogen donors. The mercury method is the first example for this general methodology. Alkylmercury hydride is generated in situ during the reaction. Shown are early syntheses of the mercury method by Kozikowski^[^ ^20^ ^]^ and by Danishefsky.^[^ ^21^ ^].^

In a second test experiment, we checked the generation of alkyl radicals from alkylalkylmercury salts by polymerization reactions. To our surprise the reaction of organomercury salts with NaBH_4_ in the presence of alkenes like acrylonitrile did not yield polymers but 1:1:1‐addition products (Scheme 2). Thus, after radical addition to acrylonitrile, the adduct‐radical is trapped faster by hydrogen atom transfer (HAT) than by acrylonitrile. This is caused by polar effects of radical addition reactions. Alkyl radicals are nucleophiles. They react preferentially with alkenes that are substituted by electron withdrawing substituents. The nitrile group at the adduct radical reduces the nucleophilicity and decreases the addition rate to acrylonitrile. The hydrogen donor is alkylmercury hydride, formed as intermediate by NaBH_4_ reaction with alkylmercury salts. HAT then leads to alkylmercury radicals that regenerate alkyl radicals. Instead of a long linear chain reaction, a three‐step cyclic chain results. Manuscripts of this new three‐component synthesis were submitted 1976 (50 years ago) to Angew. Chem. (communication)^[^ ^18^ ^]^ and to Chem. Ber. (full paper).^[^ ^19^ ^]^ Colleagues realized the interesting features of this new synthesis,^[^ ^20^, ^21^ ^]^ and 15 years later, Dennis Curran^[^ ^22^ ^]^ introduced the term “Giese reaction” for this synthetic method.

**Figure 3:**
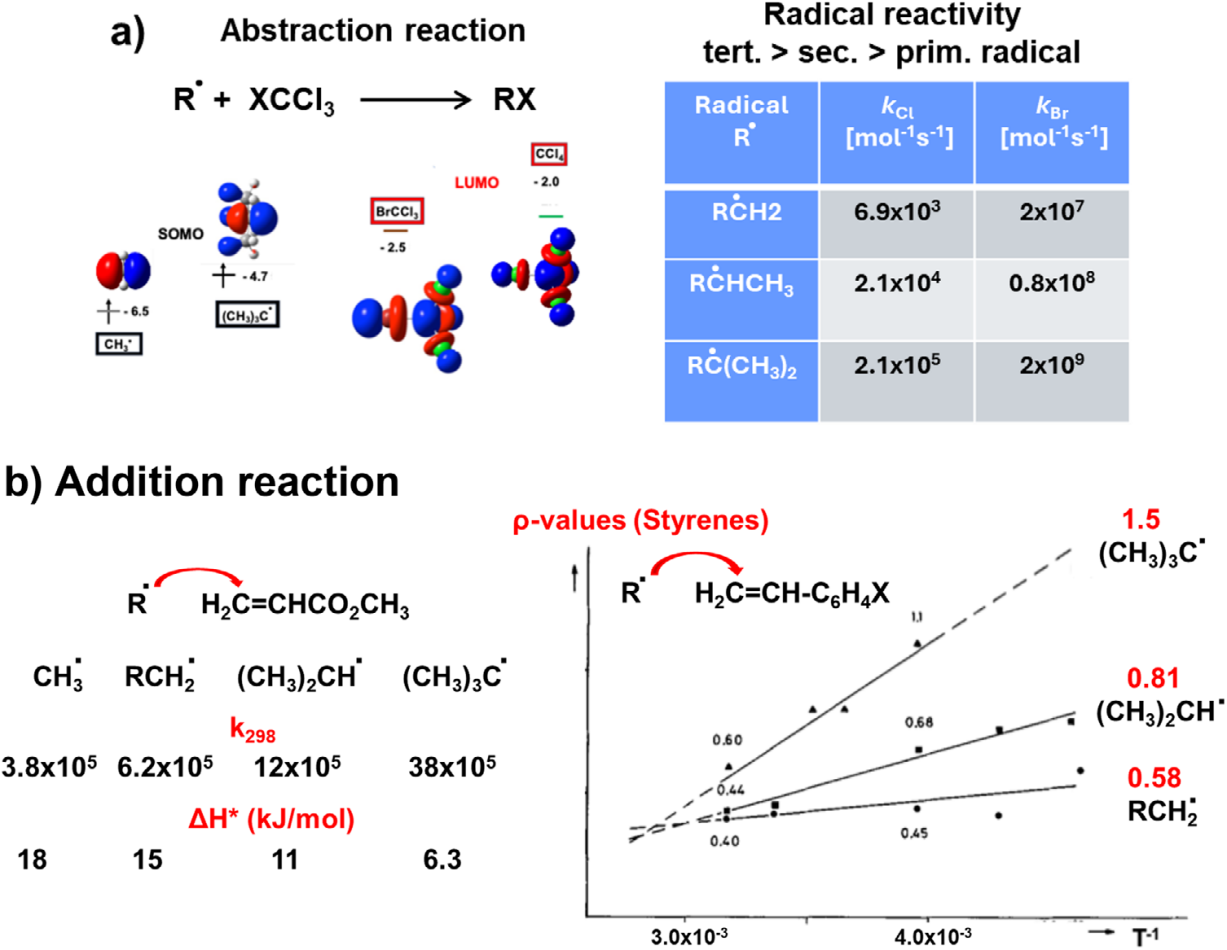
a) The rate of halogen abstraction from XCCl_3_ (X═Br, Cl) increases from prim. to *tert*. radicals, which can be explained by increasing SOMO/LUMO interactions (their energies are given in eV), b) rates of addition reactions to methyl acrylate increases from methyl to *tert*. butyl radical, and the activation enthalpies decrease as expected from the FMO theory. Below of the isoselective temperature, Hammet *ρ*‐values for addition reactions at stilbenes also increase in this order.

**Figure 4:**
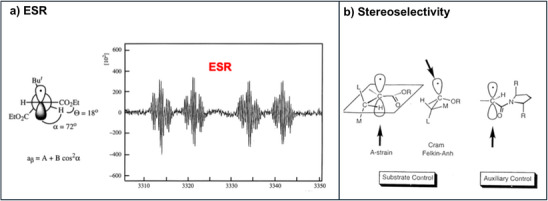
Some acyclic radicals adopt preferred conformation in their ground states, which can be determined by ESR‐spectroscopy a) From ESR coupling constants the preferred conformation is elucidated. In this conformation one face of the sp^2^‐hybridized radical is shielded by a *tert*. butyl group, which leads to steric induction of the trapping step, b) 1,2‐stereoinduction of radical reactions is determined by substrate and chiral auxiliary control. An ester group, which is in conjugation with the radical center, fixes the conformation similar to the A‐strain effect of allylic systems.

In the 1970s, multistep reactions of radicals were still written line after line, and it took until 1984 that we introduced the visualization of circular radical chain reactions as shown in Scheme 2.^[^ ^23^ ^]^ This writing became very popular and made the multistep reaction sequence understandable at a glance. Furthermore, radical chain reactions were not only used for the synthesis of target molecules but also for the elucidation of substituent effects on substrate‐, regio‐ and stereoelectivites. The results of steric, polar and mesomeric effects have been explained in several reviews^[^ ^24^ ^]^ and books.^[^ ^25^ ^]^ They shall not be described again in this personal review. We would like to mention only two effects that are not well known outside the radical community: a) in early transition states of abstraction^[^ ^9^, ^26^ ^]^ and addition reactions^[^ ^18^, ^27^ ^]^ radical rates can increase, as predicted by FMO theory (Figure 3), from prim. to tert. radicals, which can lead to an increase of selectivity by increase of reactivity;^[^ ^18^, ^28^ ^]^ b) the stereoselectivity of radical reactions follows similar rules as non‐radical reactions (Figure 4b).^[^ ^29^ ^]^

**Figure 5:**
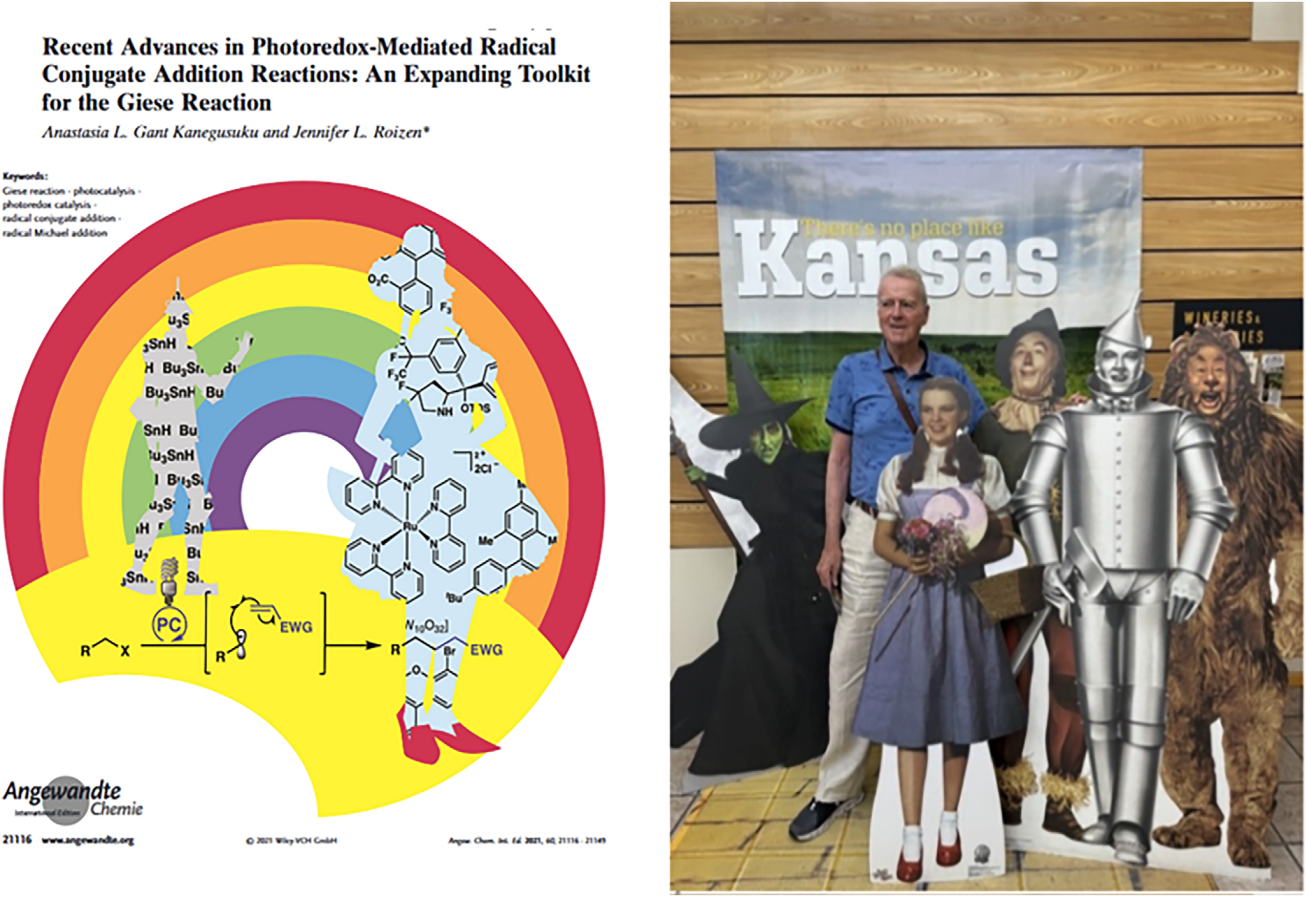
B. Giese, depicted as the “Tin Man” taken from cit. 50, and a private photo together with the real “Tin Man” of “The Wizard of Oz”.

An important aspect, which explains the success of these radical chain reactions in synthesis, is the disclosure of high stereoselectivities of cyclic^[^ ^30^, ^31^, ^32^ ^]^ as well as acyclic radicals.^[^ ^2^ ^]^ ESR‐spectroscopy is an excellent method to understand stereoselectivity^[^ ^33^, ^34^, ^35^ ^]^ because radicals are energetically close to their reaction transition states (Figure 4a). From preferred conformations of acyclic radicals by A‐strain,^[^ ^36^ ^]^ Cram–Felkin–Anh,^[^ ^34^ ^]^ and chiral auxiliary^[^ ^37^ ^]^ control the diastereoselectivity of bimolecular reactions had been established (Figure 4b). Collaboration with Dennis Curran and Ned Porter was the key to success. The results were published, inter alia, in three back‐to‐back communications,^[^ ^38^, ^39^, ^40^ ^]^ one review,^[^ ^41^ ^]^ and a monograph.^[^ ^29^ ^]^ A prediction in this monograph, written 1995, that “the development of enantioselective reactions of acyclic radicals is now inevitable” was fulfilled at the same time for cyclic^[^ ^42^ ^]^ and acyclic^[^ ^43^ ^]^ radicals. Porter and Sibi described how chiral ligands attached to the radicals by Lewis acid complexation determine enantioselectivity, and how fast background reactions can jeopardize the success (Figure 5).^[^ ^44^ ^]^

**Scheme 3:**
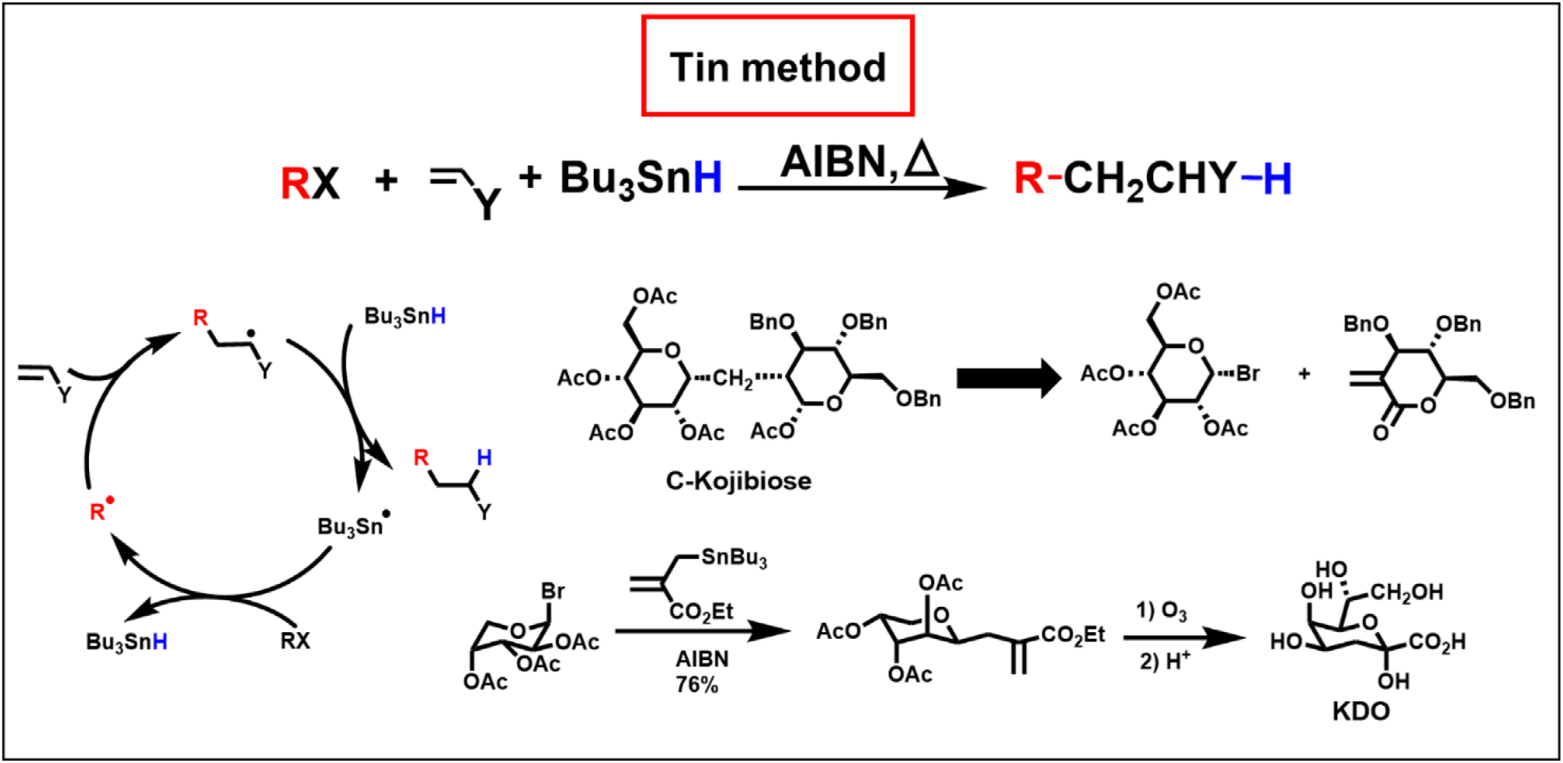
The tin method: Giese reaction using tributyltin hydride as hydrogen donor. Shown are examples for the application in carbohydrate chemistry, and its extension to allyltin educts.

A few years after the discovery of the mercury method we exchanged organomercury hydrides by tributyltin hydride, which was at the same time used by Stork for radical cyclization reactions.^[^ ^45^ ^]^ Scheme 3 depicts the three‐component synthesis with tributyltin hydride. Again, HAT is faster than the trapping of the adduct‐radical by alkenes like acrylonitrile. We applied this method especially for the synthesis of C‐glycosides,^[^ ^46^ ^]^ C‐disaccharides,^[^ ^47^ ^]^ and extended the method to chain elongation of carbohydrates, which mimics enzymatic aldol reactions with phosphoenolpyruvate.^[^ ^48^ ^]^ The tin method initiated a broad development of this multistep synthesis.^[^ ^25^, ^49^ ^]^

Today, the name “Giese reaction” is connected mainly with the tin method, although it originally started as a mercury method. In a recent review,^[^ ^50^ ^]^ Giese is thus depicted as the “Tin‐Man” of “The Wizzard of Oz”. The adjacent picture shows the scientist together with the real “Tin‐Man” of the movie.^[^ ^51^ ^]^

## 4 Selectivity and Reactivity Condition

In 1985 we proposed two conditions for successful syntheses with radicals in cyclic chain reactions: a selectivity condition and a reactivity condition.^[^ ^52^ ^]^ In cyclic chain reactions, the intermediate radicals are present during the entire synthesis, but each radical type has to react selectively with a different educt. Thus, synthesis planning is possible only if the substituent influence on radical selectivities is known. These effects had been quickly elucidated and described already in early reviews.^[^ ^24^, ^25^ ^]^ An interesting new aspect is the temperature influence on the selectivity order as discussed in chapter 2.^[^ ^9^ ^]^

**Scheme 4:**
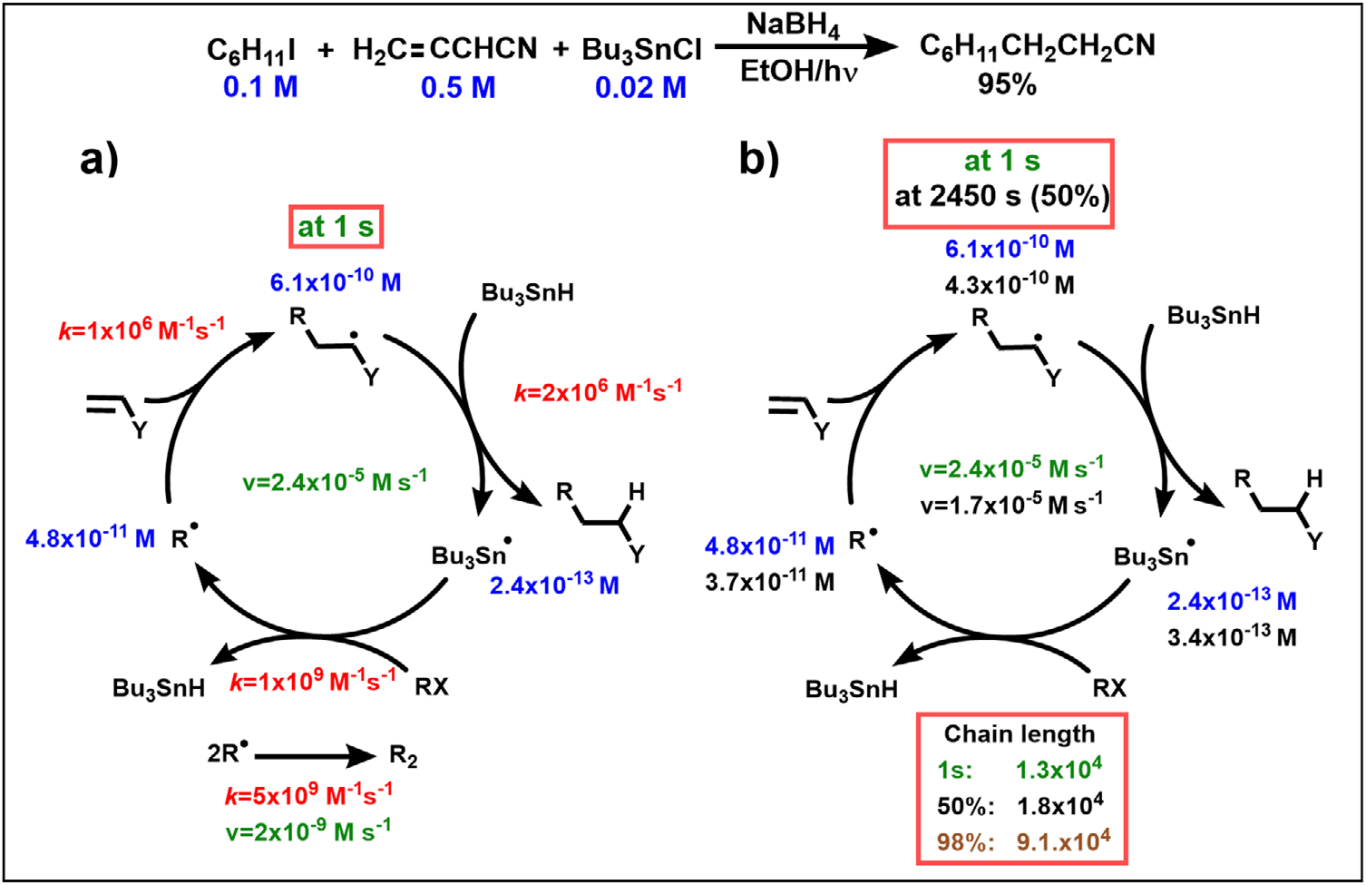
a) Rate constants *k* (red), concentrations (blue) and rates v (green) of radicals after 1s, b) influence of the conversion on radical concentrations, rates (v), and chain lengths (50% in black, 98% in brown).

New insights have also been elucidated for the second rule, the reactivity condition. As all radicals are present during the entire synthesis, chain propagation steps have to be faster than diffusion‐controlled chain terminations by radical/radical reactions. Following this rule leads to long reaction chains and reduces the formation of side‐products. A knowledge of rate constants is however not sufficient, as one also has to know the concentrations of radical intermediates. This can readily be exemplified by the photochemically initiated reaction of cyclohexyl iodide with acrylonitrile shown in Scheme 4, where the tributyltin hydride reductant is generated in situ through reduction of tributyltin halide.^[^ ^23^ ^]^ This transformation yields the 1:1:1 addition/H‐trapping product in 95% isolated yield at 25 °C, and involves the three radical chain steps shown in Scheme 4. Rate constants for alkyl radical addition to acrylonitrile,^[^ ^52^ ^]^ HAT reaction between the adduct radical and Bu_3_SnH,^[^ ^53^ ^]^ and alkyl radical generation by tributyltin radicals^[^ ^54^ ^]^ were taken or extrapolated from literature data. A fundamental requirement for a chain process to “stand” (that is, to propagate successfully with little perturbation through initiation and termination steps) is that all three chains steps proceed at identical reaction rate. The consequences of this requirement for the example shown in Scheme 4 can be tested in numerical kinetics simulations, where the concentrations of radicals and substrates are propagated in time based on the combined rate laws of all individual steps. The simulations performed by H. Zipse (SI for further details)^[^ ^55^ ^]^ show that it takes less than 1 s to reach steady state concentrations of the three chain‐carrying radicals. The reaction rates of all three chain steps are very similar at *v* = 2.4x10^−5^ M s^−1^, which is in full agreement with fundamental considerations of chain processes.^[^ ^56^ ^]^ The rate of each of the three steps depends on its rate constant (*k*), the radical concentration, and the concentration of the closed‐shell reactant. For the cyclohexyl radical addition step, for example, we have *v(add)* = *k(add)*[cyclohexyl radical][acrylonitrile]. That the reaction rates depend (numerically speaking) on the product of rate constants and radical concentrations immediately tells us, why we see comparatively high radical concentrations for steps with comparatively small rate constants (and vice versa). The concentration of tributyltin radical is lowest (at 2.4x10^−13^ M) due to its fast reaction with alkyl iodides. This is very important as trialkyltin radicals react faster with acrylonitrile than alkyl radicals,^[^ ^57^ ^]^ which could lead to unwanted side products. Another crucial result is the ten times higher concentration of the adduct radical compared to the starting alkyl radical. This ensures a faster formation of the 1:1:1‐product by HAT compared to the simple reduction of the alkyl halide. On the other hand, the addition rate constant of the adduct radical to acrylonitrile has to be much smaller than that of the nucleophilic alkyl radical. This demonstrates the importance of polar effects for successful syntheses. As expected for synthetically efficient chain reactions,^[^ ^56^ ^]^ the rates of the three chain steps are about 10^4^ times faster than chain terminating radical/radical reactions, which is a remarkable feature for the synthetic method (Scheme 4a). The chain length even increases during the reaction, because the concentrations of the carbon‐centered radicals decrease (Scheme 4b). Nevertheless, the ratio of the carbon‐centered radicals changes only slightly from 12.7 (after 1 s) to 11.6 (at 50% conversion) and 10.2 (at 98% conversion). This kinetic analysis might explain why the “Giese reaction” is so stable that it could stimulate the design of many superb new methods.^[^ ^25^, ^49^ ^]^

## 5 Redox Synthesis

**Scheme 5:**
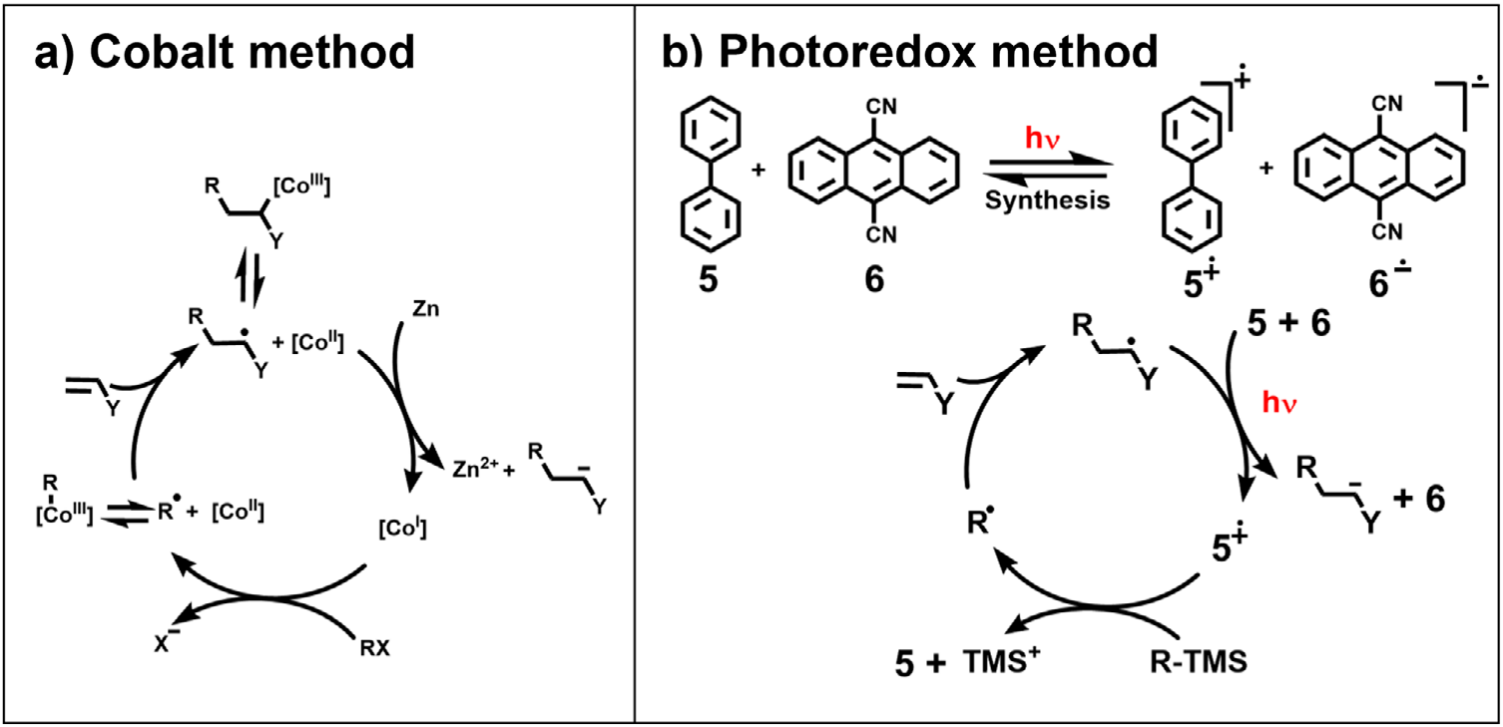
Trapping of adduct radicals by ET, a) Co‐method, b) photoredox‐method.

Already in the 1980s, methods were developed in which electron transfer (ET) instead of hydrogen atom transfer (HAT) trapped the adduct radicals. Scheffold used Vit B_12_ under reducing conditions, but the radical nature of the synthesis remained unclear.^[^ ^58^ ^]^ We applied cobaloximes for synthetic purposes,^[^ ^59^ ^]^ and elucidated a radical mechanism by comparing the cobalt‐method with the previously established mercury‐ and tin‐methods (Scheme 5a).^[^ ^60^ ^]^ As cobalt changes its oxidation state reversibly between Co(III) and Co(I), the cobalt‐method can be described as a sequence of redox‐reactions. A photo‐redox synthesis of the “Giese reaction”, where both the starting radicals are generated and the adduct radicals are trapped by ET, was developed in1995 by Eric Meggers in his diploma work (Scheme 5b).^[^ ^61^ ^]^

A photoredox synthesis that is based on radical addition to olefins and trapping of adduct radicals by HAT was to our knowledge first published in 1991.^[^ ^62^ ^]^ The huge importance and fast development of general and broad photoredox syntheses, boosted by the seminal work of McMillan,^[^ ^63^ ^]^ is not part of this personal minireview on the “Giese reaction”, but it is a major reason for the second renaissance of radical chemistry, described by Stephenson, Studer and Curran in 2013.^[^ ^64^ ^]^ As a consequence, our first review on the new radical three‐component reaction, written 1983, was cited 67 times in the year 2024 – one generation after its publication.^[^ ^24^ ^]^ A further push for our work came 1966 from microbiology, as explained in the following chapters.

## 6 Enzymatic Synthesis

**Scheme 6:**
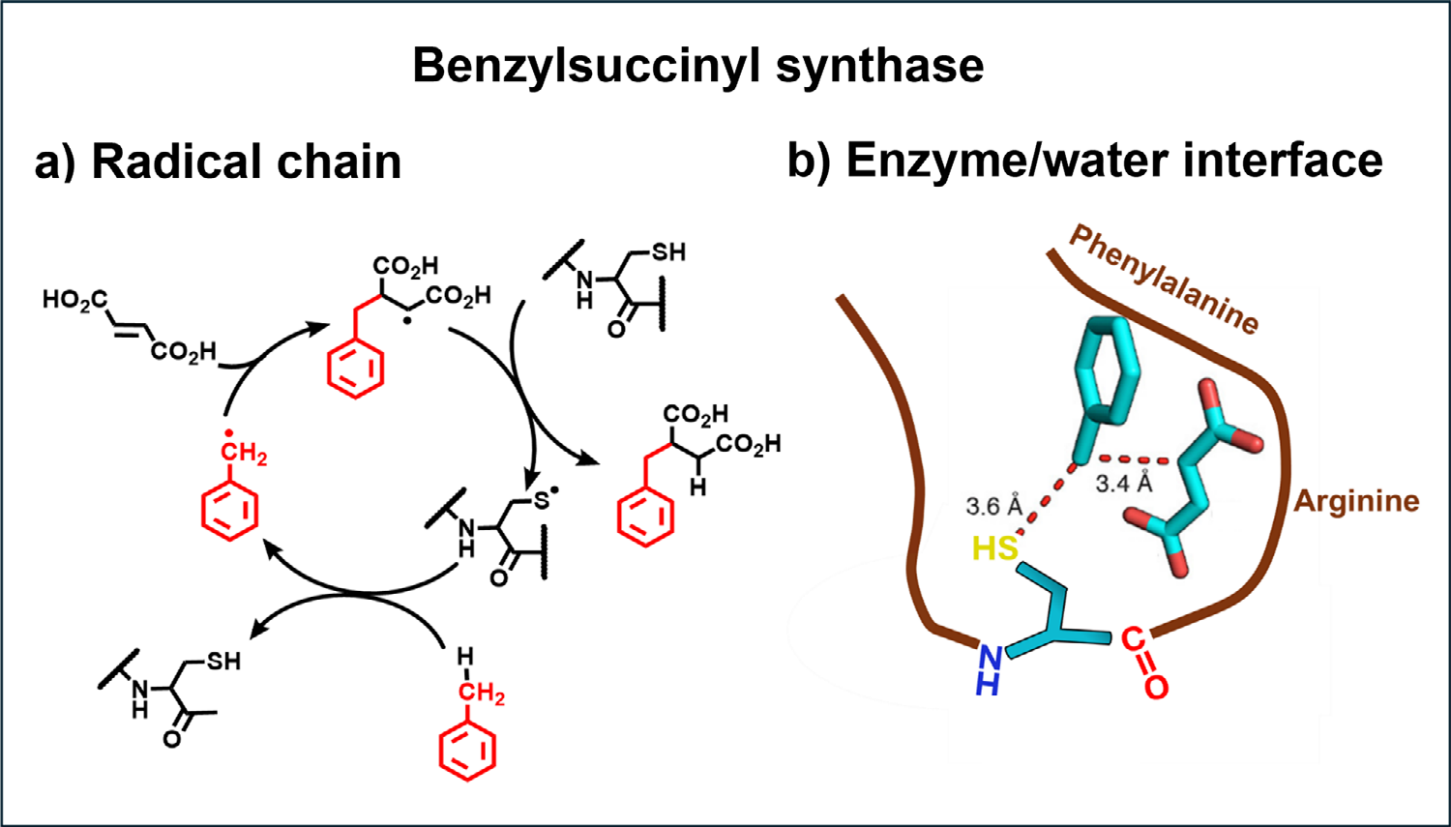
a) Enzymatic three‐component cyclic chain reaction with cysteine as hydrogen atom donor and cysteinyl radicals as precursors for benzyl radicals that add to fumarate, b) situation at the water/protein interface. Interactions with amino acids of the enzyme place educts close to each other and pave the way for the enantioselective synthesis.

The entry of the Giese‐type reaction into microbiology was the discovery in 1966 that *Thauera aromatica* bacteria catalyze the addition of toluene to fumarate in a three‐component radical synthesis (Scheme 6a).^[^ ^65^ ^]^ Adduct radicals are trapped by cysteine, and the cysteinyl radicals generate benzyl radicals by hydrogen abstraction from toluene. This is a surprising sequence. Thiyl radicals are intermediates of click reactions with alkenes,^[^ ^66^ ^]^ and they add faster to fumarate than they abstract H‐atoms from toluene.^[^ ^67^, ^68^ ^]^ Thus, the enzymatic synthesis does not follow the selectivity condition, which controls reactions in homogeneous solution. This is because enzymatic reactions proceed at the water/enzyme interface where amino acids of the enzyme hold educts at short distances that pave the way for the reaction. The X‐ray structure by C. Drennan^[^ ^69^ ^]^ demonstrates that reaction educts in the pocket of the chiral benzylsuccinyl synthase are oriented to each other as shown in Scheme 6b. Consequently, the benzyl radical addition to fumarate yields enantioselectively R‐benzylsuccinate. By applying and modifying enzymes to organic synthesis, new enantioselective methodologies with radicals are developed these days. Outstanding examples are intramolecular C,C‐bond forming reactions of radicals, generated by metal‐catalyzed hydrogen transfer,^[^ ^70^ ^]^ and photoinduced intermolecular syntheses including the Giese‐type reaction.^[^ ^71^, ^72^, ^73^ ^]^ This is a fascinating new area. Biological radical reactions show another feature: there is only one radical present at a time in the enzyme pocket, so that radical/radical recombinations cannot occur. Thus, also the reactivity condition of reactions in homogeneous solution does not play a role in enzymatic reactions.

**Figure 6:**
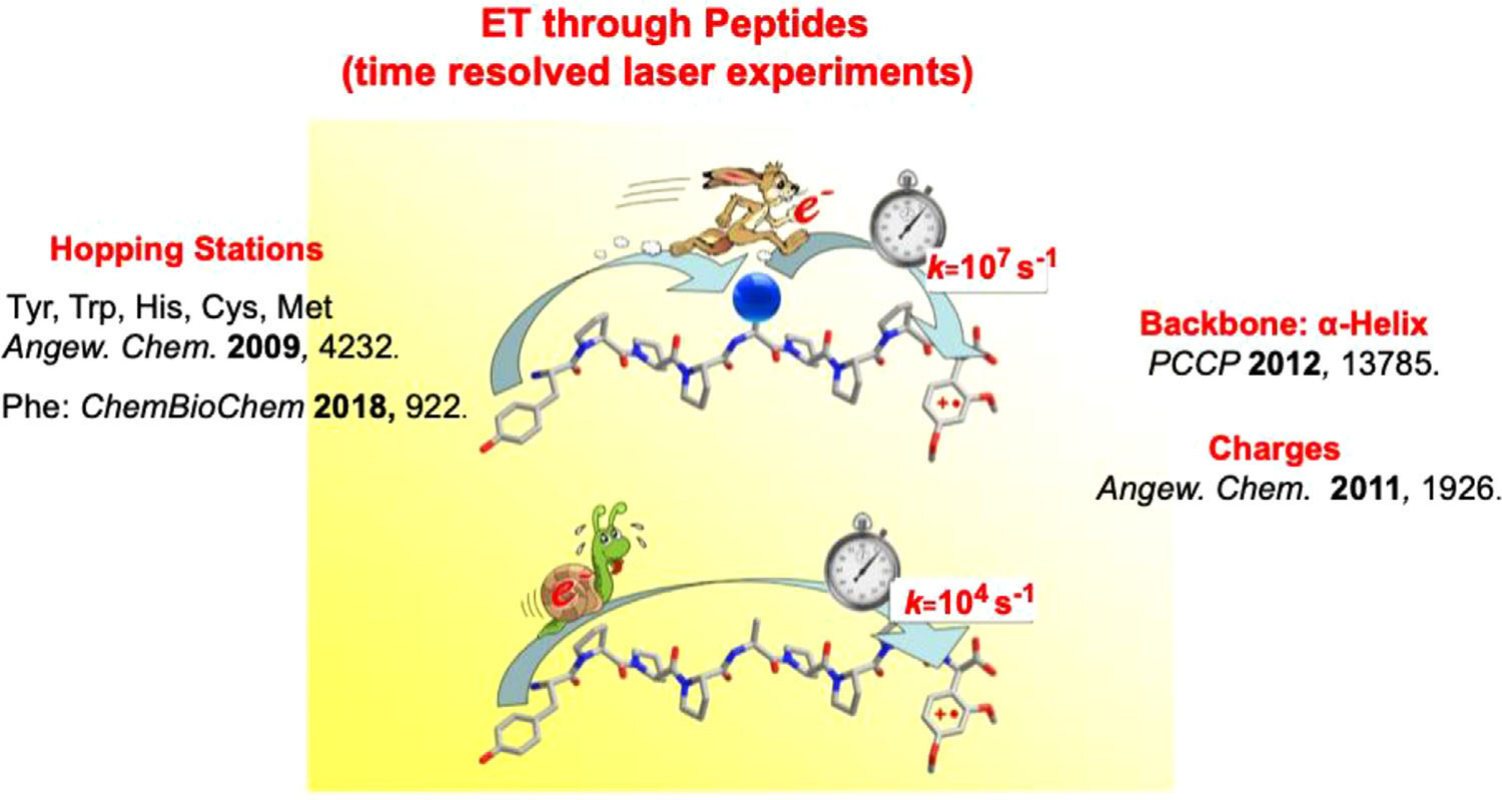
ET through peptides use aromatic and sulfur containing amino acids as hopping stations. This enables long‐distance ET in biological systems. ET rates also depend upon sec. structures and charges. The picture is in part taken from the frontispiece of cit. 82.

The generation of amino acid radicals in typical enzymatic reactions uses metallo‐cofactors. They can oxidize even distant amino acids in multistep electron (hole) hopping reactions. For benzylsuccinyl synthase a [4Fe‐4S]^2+^ cluster, positioned far away from the site of organic synthesis, oxidizes glycine at the interface.^[^ ^69^, ^74^ ^]^ The resulting glycinyl radical generates a cysteinyl radical, which drives the cyclic chain shown in Scheme 6. In the enzyme ribonucleotide reductase,^[^ ^75^, ^76^ ^]^ iron‐ and manganese‐cofactors are the oxidants.^[^ ^77^, ^78^ ^]^ They first produce a nearby tyrosine radical that oxidizes cysteine at the interface. The distance between these two amino acids is with 32 Å longer than the supposed limit of 20 Å for single‐step ET in proteins.^[^ ^79^ ^]^ Such long‐distance ET can occur only in a multistep process.^[^ ^80^ ^]^ Our time‐resolved laser experiments showed that aromatic and sulfur‐containing amino acids are hopping stations, which break down one long ET into several short ET steps (Figure 6).^[^ ^81^, ^82^, ^83^ ^]^ The mechanism is similar to ET through double‐stranded DNA where heteroaromatic nucleotides are hopping stations, and ET follows diffusion rules.^[^ ^84^, ^85^, ^86^, ^87^ ^]^

**Figure 7:**
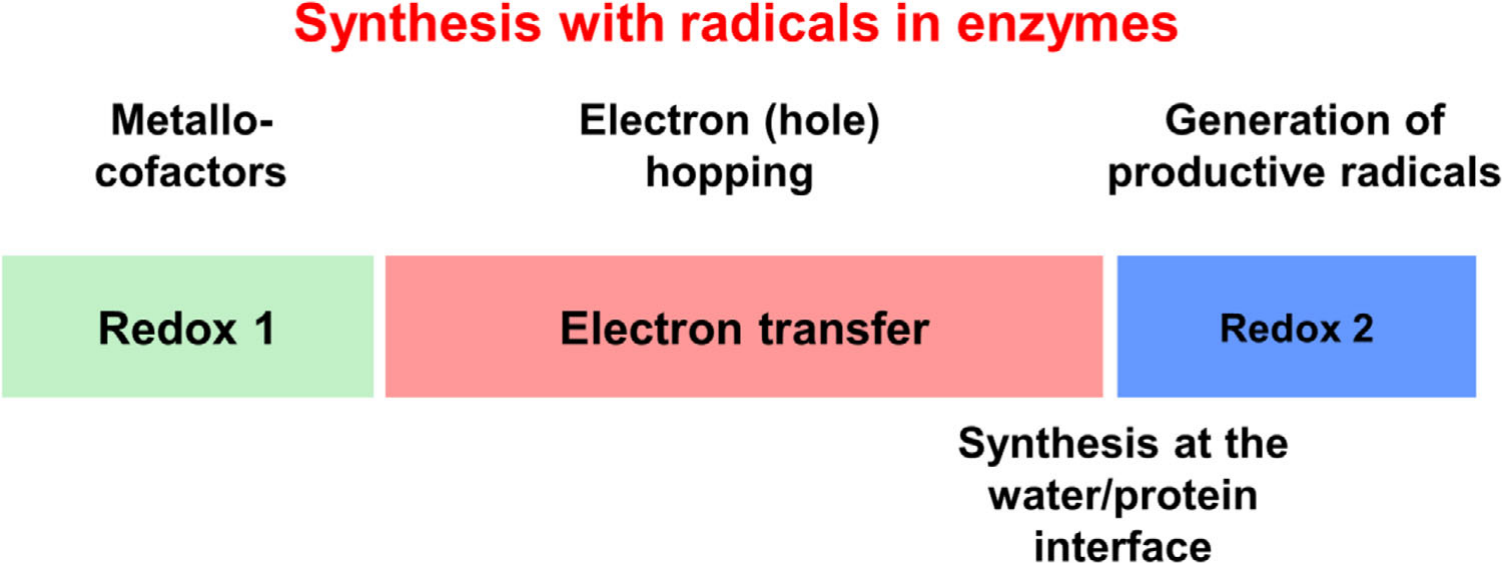
Typical set‐up of enzymatic redox reactions. A metallo‐cofactor produces productive radicals by long‐distance ET. Synthesis occurs at the protein/water interface.

Thus, many radical and redox reactions in enzymes and biological cells follow the 3‐step process depicted in Figure 7: a) or redox active metallo‐cofactor is positioned far away from the synthesis site, b) long‐distance ET in a multistep hopping process yields the productive radical, and c) product synthesis proceeds at the water/protein interface. We have measured kinetics of the three different processes in living cells.

## 7 Cellular Reactions

**Figure 8:**
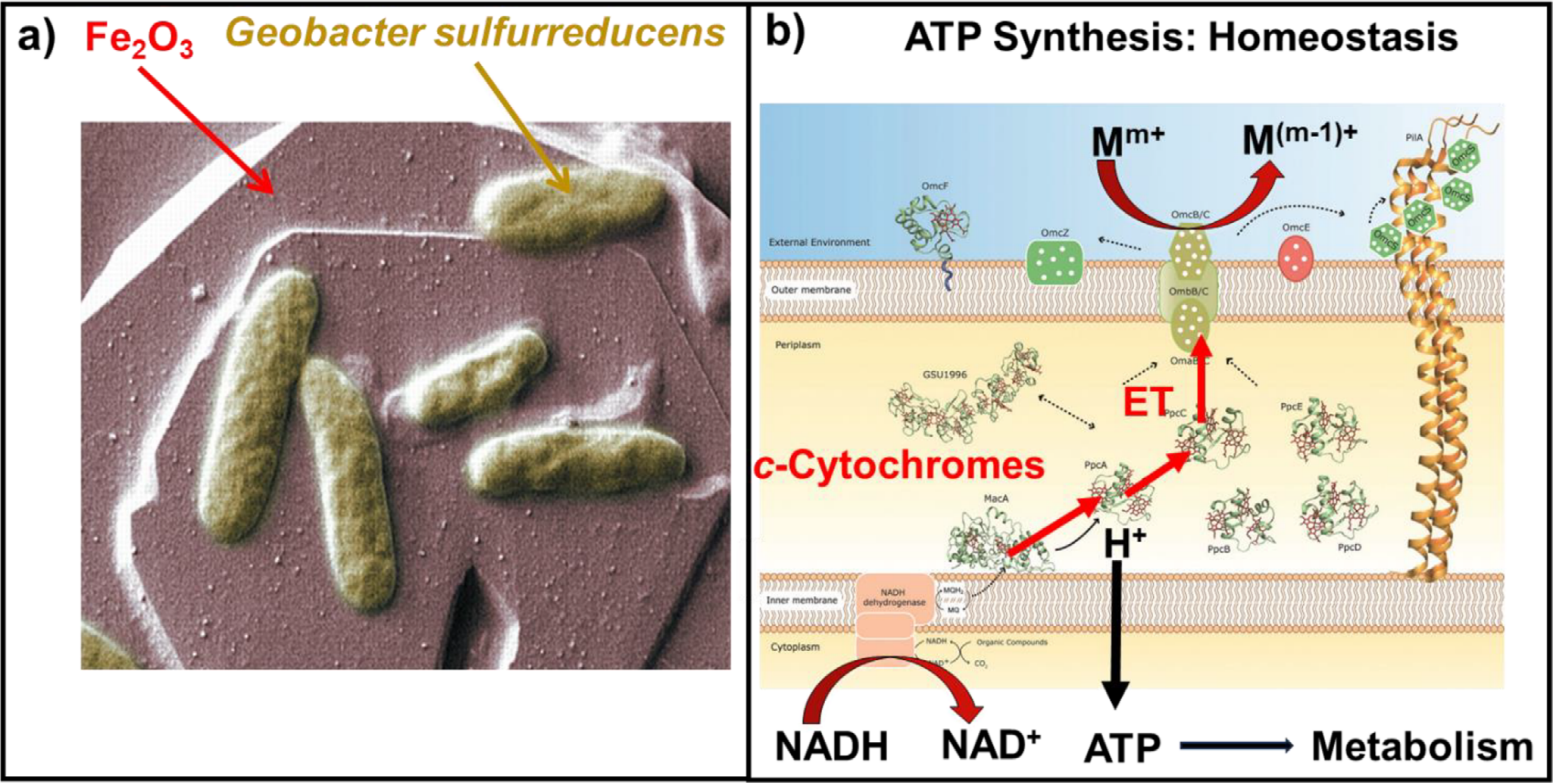
a) *Geobacter sulfurreducens* is attached to an oxidizing mineral, that accepts electrons from reductive equivalents of the cell, b) extracellular metal ions at the outer membrane oxidize the cytoplasmic NADH at the inner membrane. Electron transport from the inner membrane through the periplasm to the outer membrane is mediated by multiheme cytochromes in the periplasm. The proton gradient activates ATPase to synthesize ATP, driving the metabolism and cell growth. The vitality of the cells confirms ATP‐homeostasis. On the right, a bundle of proteins (pili) is shown that might play a role in ET under certain reaction conditions.

The work‐horse that we have used to measure rates and reaction orders of the 3‐step process, shown in Figure 7, is *Geobacter sulfurreducens* (Figure 8a).^[^ ^88^, ^89^ ^]^ These anaerobic microorganisms can utilize extracellular metal salts as oxidants for their respiration.^[^ ^90^ ^]^ The 3‐step process starts with oxidation of the Fe^2+^/hemes of the outer membrane cytochromes (OMCs), which is followed by long distance ET through the periplasmic *c*‐cytochromes, and finally the Fe^3+^/hemes at the inner membrane oxidize NADH. Proton‐coupled ET stimulates ATP synthesis by ATPase (Figure 8b).

**Figure 9:**
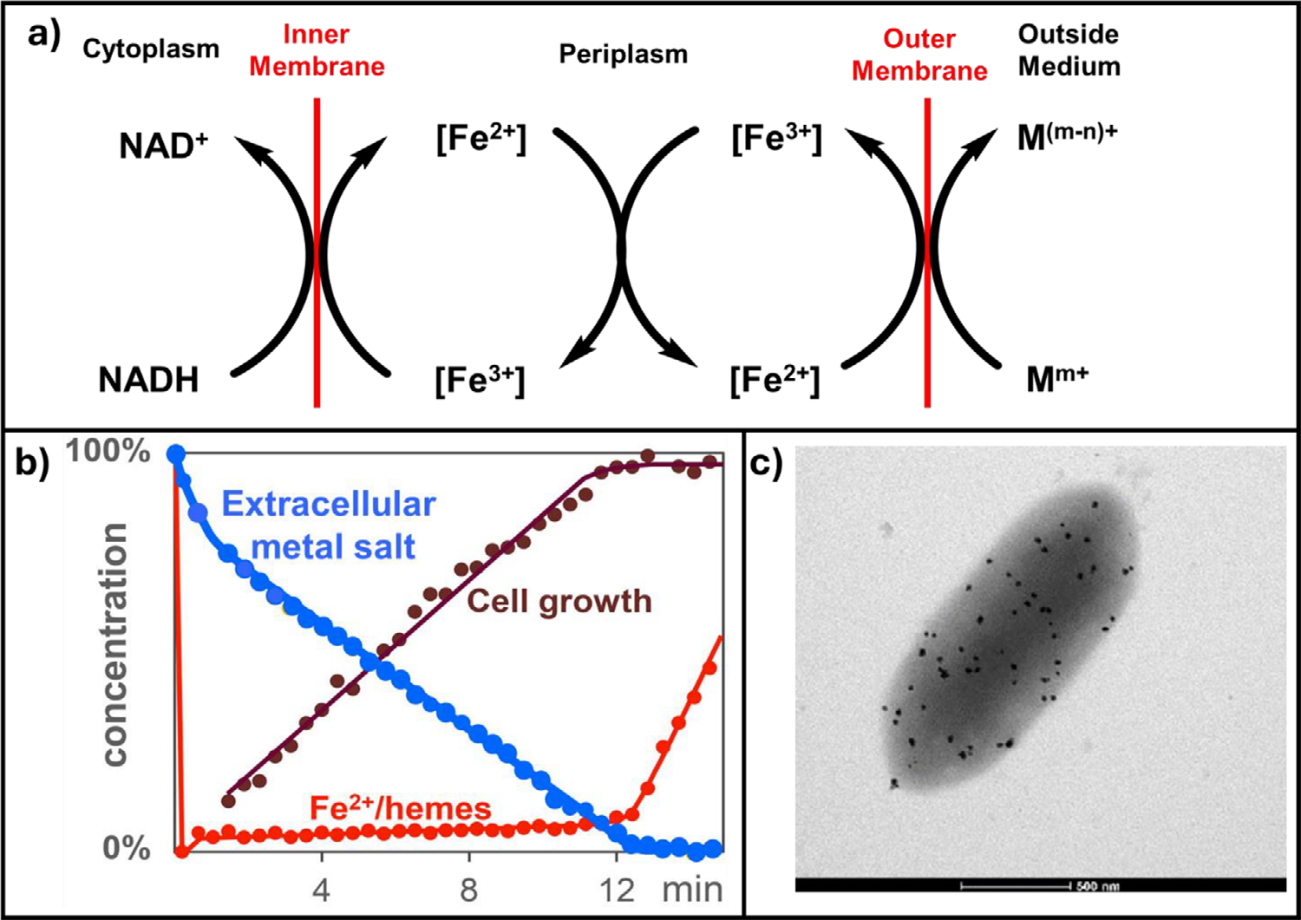
a) Sketch of the *Geobacter sulfurreducens* respiration as an example for the 3‐step process of redox reactions in cells. Cytoplasmic NADH at the inner membrane is synthesized by extracellular metal salts at the outer membrane. Electron transport between the reactants occurs in multistep ET processes between the periplasmic *c*‐cytochromes. b) Concentration changes of the oxidizing, extracellular metal ions (blue line), Fe^2+^/hemes of the cytochromes (red line), and cells (brown line), c) Ag‐nanoparticles synthesized at *Geobacter sulfurreducens* during respiration with AgNO_3_, which makes the number of outer membrane cytochromes visible.

We studied this process by measuring a) kinetics of the metal salt reduction at the outer membrane, b) ET through the periplasm by observing [Fe^2+^]/[Fe^3+^] changes of the *c*‐cytochromes, and c) rates of ATP consumption by cell growth (Figure 9b).^[^ ^88^, ^89^ ^]^ The surprising result is that reduction of metal salt oxidants occurs with zero‐order kinetics (blue line in Figure 9b). That is, the chemical reaction is independent of the metal salt concentration. This discloses a characteristic of interface reactions: as the number of reaction sites (OMCs in Figure 8b) is constant, and their quantity, determined by Ag‐nanoparticle formation at the cell surface with AgNO_3_ as oxidant, is small (Figure 9c) the metal salt reduction proceeds in a saturated Michaelis‐Menten reaction. A second important feature is shown by the electron transporting *c*‐cytochromes. They regulate the rate of the respiration process. In the resting state of the bacteria, all iron‐hemes contain Fe^2+^ ions. Addition of metal salts oxidize them in less than a second to Fe^3+^/hemes (red line in Figure 9b), which induces respiration by NADH oxidation. During respiration, the Fe^2+^/Fe^3+^ ratio remains nearly constant, demonstrating a constant long‐distance ET rate through the cell. After the oxidant is consumed, the remaining Fe^3+^ ions are reduced by NADH, and the bacteria reach the resting state again (red trace in Figure 9b).^[^ ^88^, ^89^ ^]^

Metal salt reduction (blue line in Figure 9b) that leads to ATP on one hand and cell growth (brown line in Figure 9b) that consumes ATP on the other follow the same constant concentration change. This confirms the existence of ATP‐homeostasis, a condition for a viable metabolism. Further experiments had shown that ATP‐homeostasis remains stable even if reaction conditions change.^[^ ^89^ ^]^ The oxidation effect of different metal salts as well as changes of *c*‐cytochrome concentrations, induced by mutations or switching growth conditions, are regulated by multiheme *c*‐cytochromes. They adjust their redox potentials by changing the Fe^2+^/Fe^3+^ equilibrium so that the reaction rates and with this the ATP synthesis, remains constant. This demonstrates the decisive function of *c*‐cytochromes as “rate buffers”. It is fascinating to see that biological ET can increase from several nanometers through enzymes^[^ ^75^, ^76^ ^]^ via micrometers with multi‐protein filaments (pili, see Figure 8b)^[^ ^91^ ^]^ to centimeters in multi‐cellular cable bacteria.^[^ ^92^ ^]^ The role of metal ions for long‐distance ET processes through pili^[^ ^93^, ^94^, ^95^ ^]^ and cable bacteria^[^ ^96^, ^97^, ^98^ ^]^ is still under study. Another interesting question is: are these pili and cable bacteria not only electrical switches but also rate regulators like *c*‐cytochromes in *Geobacter sulfurreducens*?

## 8 Conclusion

Studies of Giese‐type radical synthesis in homogeneous solution (laboratory vessel) and under heterogeneous conditions (biological cells) recall the typical features of two different experimental set‐ups. Reactions in homogeneous solution are driven by kinetic effects of mobile molecules. A successful synthesis in the laboratory vessel can be described as a solution of a kinetic problem (chapters 2–4). This is in strong contrast to analogous biological reactions (chapter 6). Here, non‐covalent binding forces of amino acids at the water/protein interface bring educts in close contact to each other, which paves the way for their reactions. Thus, in enzymes thermodynamic effects are more important. Redox reactions in cells, discussed in chapter 7, demonstrate a consequence of this behavior: educt concentration changes during the reaction, that is kinetics, nearly don't play a role in product synthesis. This is caused by the limited and constant number of reaction sites at the interface, which can lead to a saturated Michaelis‐Menten behavior. There is another feature of enzymatic and cellular radical/redox reactions: active molecules are often generated far away from the interface, at which the turnover takes place. Multiheme *c*‐cytochromes as mediators for ET between these sites buffer synthesis rates just by changing the Fe^2+^/Fe^3+^ ratio (chapter 7). The challenge for a chemist remains to harness analogous regulation effects for organic syntheses in the laboratory.

## Supporting Information

The Supporting Information describes details for the kinetic date of chapter 4.

## Conflict of Interests

The authors declare no conflict of interest.

## Supporting information



Supporting Information

## Data Availability

The data that support the findings of this study are available in the Supporting Information of this article.

## References

[1] L. Kaplan , Bridged Free Radicals, Marcel Dekker, New York, 1972.

[2] T. Kawamura , D. J. Edche , J. K. Kochi , J. Am. Chem. Soc. 1972, 94, 1752–1754, 10.1021/ja00760a060.

[3] A. R. Lyons , M. C. R. Symons , J. Am. Chem Soc. 1971, 93, 7330–7331, 10.1021/ja00755a043.

[4] B. Giese , Chem. Ber. 1974, 107, 819–831, 10.1002/cber.19741070308.

[5] C. L. Hill , G. M. Whitesides , J. Am. Chem Soc. 1974, 96, 870.

[6] B. Giese , Angew. Chem. Int. Ed. Engl. 1976, 15, 173–174, 10.1002/anie.197601731.

[7] L. Liu , Q.‐X. Guo , Chem. Rev. 2001, 101, 673–696, 10.1021/cr990416z. 11712500

[8] B. Giese , Angew. Chem. Int. Ed. Engl. 1977, 16, 125–136, 10.1002/anie.197701253.

[9] A. Kern , M. Spichty , B. Giese , submitted.

[10] T. Clark , J. Phys. Chem. A 2019, 123, 3326–3333, 10.1021/acs.jpca.9b01133. 30916961

[11] P. M. W. Gill , L. Radom , J. Am. Chem. Soc. 1988, 110, 4931–4941, 10.1021/ja00223a010.

[12] K. V. Macken , H. W. Sidebottom , Int. J. Chem. Kin. 1979, 11, 511–527, 10.1002/kin.550110505.

[13] J. D. Dunitz , Chem. Biol. 1995, 2, 709. 9383477 10.1016/1074-5521(95)90097-7

[14] J. M. Fox , M. Zhao , M. J. Fink , K. Kang , G. M. Whitesides , Ann. Rev. Biophys. 2018, 47, 223–250, 10.1146/annurev-biophys-070816-033743. 29505727

[15] U. Ryde , MedChemComm 2014, 5, 1324–1336, 10.1039/C4MD00057A.

[16] F. Peccati , G. Jiménez‐Osés , ACS Omega 2021, 6, 11122–11130, 10.1021/acsomega.1c00485. 34056267 PMC8153931

[17] K. N. Houk , A. G. Leach , S. P. Kim , X. Zhang , Angew. Chem. Int. Ed. Engl. 2003, 42, 4872–4897, 10.1002/anie.200200565. 14579432

[18] B. Giese , J. Meister , Angew. Chem. Int. Ed. Engl. 1977, 10, 178.

[19] B. Giese , J. Meister , Chem. Ber. 1977, 110, 2588–2600, 10.1002/cber.19771100717.

[20] A. P. Kozikowski , T. R. Niduzak , J. Sripko , Organometallics 1982, 1, 675–676, 10.1021/om00064a018.

[21] S. Danishefsky , E. Taniyama , R. R. Webb , Tetrahedron Lett. 1983, 24, 11–14, 10.1016/S0040-4039(00)81313-1.

[22] C. P. Jasperse , D. P. Curran , T. L. Flevig , Chem. Rev. 1991, 91, 1237–1286, 10.1021/cr00006a006.

[23] B. Giese , J. A. Gonzalez‐Gomez , T. Witzel , Angew. Chem. Int. Ed. Engl. 1984, 23, 69.

[24] First review: B. Giese , Angew. Chem. Int. Ed. Engl. 1983, 22, 753–764, 10.1002/anie.198307531.

[25] B. Giese , Radicals in organic synthesis: formation of carbon‐carbon bonds, Pergamon Press, Oxford 1986.

[26] B. Giese , J. Hartung , Chem. Ber. 1992, 125, 1777–1779, 10.1002/cber.19921250734.

[27] H. Fischer , L. Radom , Angew. Chem. Int. Ed. Engl. 2001, 40, 1340–1371, 10.1002/1521-3773(20010417)40:8<1340::AID-ANIE1340>3.0.CO;2-. 11317286

[28] B. Giese , J. Meixner , Angew. Chem. Int. Ed. Engl. 1979, 18, 154–155, 10.1002/anie.197901541.

[29] D. P. Curran , N. A. Porter , B. Giese , Stereochemistry of Radical Reactions, CCH, Weinheim 1996.

[30] B. Giese , K. Heuck , H. Lenhardt , U. Lüning , Chem. Ber. 1984, 117, 2132–2139, 10.1002/cber.19841170610.

[31] B. Giese , Angew. Chem. Int. Ed. Engl. 1989, 28, 969–980, 10.1002/anie.198909693.

[32] W. Damm , B. Giese , J. Hartung , T. Hasskerl , K. N. Houk , H. Zipse , J. Am. Chem. Soc. 1992, 114, 4067–4079, 10.1021/ja00037a007.

[33] B. Giese , W. Damm , F. Wetterich , H. G. Zeitz , Tetrahedron Lett. 1992, 33, 1863–1866, 10.1016/S0040-4039(00)74162-1.

[34] B. Giese , W. Damm , J. Dickhaut , F. Wetterich , S. Sun , D. P. Curran , Tetrahedron. Lett. 1991, 32, 6097–6100, 10.1016/0040-4039(91)80762-U.

[35] G. Thoma , D. P. Curran , S. V. Geib , B. Giese , W. Damm , F. Wetterich , J. Am. Chem. Soc. 1993, 115, 8585–8591, 10.1021/ja00072a010.

[36] B. Giese , M. Bulliard , H. G. Zeitz , Synlett 1991, 1991, 425–427, 10.1055/s-1991-20751.

[37] N. A. Porter , D. M. Scott , B. Lacher , B. Giese , H. G. Zeitz , H. J. Lindner , J. Am. Chem Soc. 1989, 111, 8311–8312, 10.1021/ja00203a060.

[38] D. P. Curran , W. Shen , J. Zhang , T. A. Heffner , J. Am. Chem. Soc 1990, 112, 6738–6740, 10.1021/ja00174a059.

[39] N. A. Porter , E. Swann , J. Nally , A. T. McPhall , J. Am. Chem. Soc. 1990, 112, 6740–6741, 10.1021/ja00174a060.

[40] B. Giese , M. Zehnder , M. Roth , H. G. Zeitz , J. Am. Chem. Soc 1990, 112, 6741–6742 10.1021/ja00174a061.

[41] N. A. Porter , B. Giese , D. P. Curran , Acc. Chem. Res. 1991, 24, 296–304, 10.1021/ar00010a003.

[42] M. Murakata , H. Tsutsui , O. Hishono , J. Chem. Soc. Chem. Commun. 1995, 481–482.

[43] J. H. Wu , R. Radinov , N. A. Porter , N. A. J. Am. Chem. Soc. 1995, 117, 11029–11030, 10.1021/ja00149a035.

[44] M. Sibi , N. A. Porter , Acc. Chem. Res. 1999, 32, 163–171, 10.1021/ar9600547.

[45] G. Stork , N. H. Baine , J. Am. Chem. Soc. 1982, 104, 2321–2323, 10.1021/ja00372a042.

[46] B. Giese , J. Dupuy , Angew. Chem. Int. Ed. Engl. 1983, 22, 622–623, 10.1002/anie.198306221.

[47] B. Giese , M. Hoch , C. Lamberth , R. R. Schmidt , Tetrahedron Lett. 1988, 29, 1375–1378, 10.1016/S0040-4039(00)80300-7.

[48] B. Giese , T. Linker , R. Muhn , Tetrahedron 1989, 45, 935–940, 10.1016/0040-4020(89)80005-5.

[49] P. Renauld , M. Sibi , Radicals on Organic Synthesis, Wiley‐VCH, Weinheim 2001.

[50] A. L. G. Kanegusuku , J. L. Roizen , Angew. Chem. Int. Ed. Engl. 2021, 60, 21149.

[51] One reviewer remarked: “I only wonder whether Giese would prefer to be called Mercury Man rather than Tin Man. The Tin Man is a rather goofy character who ultimately got a heart. In contrast, Mercury Man is a superhero” in the movie of the same name.

[52] B. Giese , Angew. Chem. Int. Ed. 1985, 24, 553–565, 10.1002/anie.198505531.

[53] L. J. Johnston , J. Lusztyk , D. D. M. Wayner , A. N. Abeywickreyma , A. L. J. Beckwith , J. C. Scaiano , K. U. Ingold , J. Am. Chem. Soc. 1985, 107, 4594–4596, 10.1021/ja00301a062.

[54] F. Julia , T. Constantin , D. Leonori , Chem. Rev. 2022, 122, 2292–2352, 10.1021/acs.chemrev.1c00558. 34882396

[55] COPASI 4.44 (Build 295). COPASI is a simulator for biochemical networks accessible at https://copasi.org/. It is a joint project by the Hoops group (Biocomplexity Institute of Virginia Tech), the Mendes group (UCONN School of Medicine), the Kummer, and Sahle groups (University of Heidelberg).

[56] M. Newcomb , Tetrahedron 1993, 49, 1151–1176, 10.1016/S0040-4020(01)85808-7.

[57] C. Chatgilialoglu , K. U. Ingold , J. C. Scaiano , J. Am. Chem. Soc. 1983, 105, 3292–3296, 10.1021/ja00348a055.

[58] R. Scheffold , G. Rytz , L. Walder , R. Orlinski , Z. Chilmonczyk , Pure & Appl. Chem. 1983, 55, 1791–1797, 10.1351/pac198355111791.

[59] A. Ghosez , T. Göbel , B. Giese , Chem. Ber. 1988, 121, 1807–1811, 10.1002/cber.19881211018.

[60] B. Giese , J. Hartung , J. He , O. Hüter , A. Koch , Angew. Chem. Int. Ed. Engl. 1989, 28, 325.

[61] E. Meggers , E. Steckhan , S. Blechert , Angew. Chem. Int. Ed. Engl. 1995, 34, 2137–2139, 10.1002/anie.199521371.

[62] K. Okada , K. Okamoto , N. Morita , K. Okubo , M. Oda , J. Am. Chem. Soc. 1991, 113, 9401–9402, 10.1021/ja00024a074.

[63] M. H. Shaw , J. Twilton , D. W. C. MacMillan , J. Org. Chem. 2016, 81, 6898–6926, 10.1021/acs.joc.6b01449. 27477076 PMC4994065

[64] C. R. Stephenson , A. Studer , D. P. Curran , Beilstein J. Org. Chem. 2013, 9, 2778–2780, 10.3762/bjoc.9.312. 24367441 PMC3869312

[65] T. Biegert , G. Fuchs , J. Heider , Eur. J. Biochem. 1996, 238, 661–668, 10.1111/j.1432-1033.1996.0661w.x. 8706665

[66] A. Dondoni , Angew. Chem. In. Ed. Engl 2008, 47, 8995–8997, 10.1002/anie.200802516. 18846513

[67] B. H. Northorp , R. N. Coffey , J. Amer. Chem. Soc. 2012, 134, 13804–13817, 10.1021/ja305441d. 22853003

[68] A. G. Vandeputte , M.‐F. Reyniers , G. B. Marin , ChemPhysChem 2013, 14, 3751–3771, 10.1002/cphc.201300661. 24590616

[69] M. A. Funk , E. N. G. Marsh , C. L. Drennan , J. Biol. Chem. 2015, 290, 22398–22408, 10.1074/jbc.M115.670737. 26224635 PMC4566215

[70] D. Chen , X. Zhang , A. A. Vorobieva , R. Tachibana , A.l. Stein , R. P. Jakob , Z. Zou , D. A. Graf , A. Li , T. Maier , B. E. Correia , T. R. Ward , Nature Chem 2024, 16, 1656–1664, 10.1038/s41557-024-01562-5. 39030420

[71] X. Huang , J. Feng , J. Cui , G. Jiang , W. Harrison , X. Zang , J. Zhou , B. Wang , H. Zhao , Nature Catal 2022, 5, 586–593 10.1038/s41929-022-00777-4.

[72] M. A. Emmanuel , S. G. Bender , C. Bilodeau , J. M. Carceller , J. S. DeHovitz , H. Fu , Y. Liu , B. T. Nicholls , Y. Ouyang , C. G. Page , T. Qiao , F. C. Raps , D. R. Sorigué , S.‐Z. Sun , J. Turek‐Herman , Y. Ye , A. Rivas‐Souchet , J. Cao , T. K. Hyster , Chem. Rev. 2023, 123, 5459–5520, 10.1021/acs.chemrev.2c00767. 37115521 PMC10905417

[73] V. Tseliou , L. Kqiku , M. Berger , F. Schiel , H. Zhou , G. J. Poelarends , P. Melchiorre , Nature 2024, 634, 848–854, 10.1038/s41586-024-08004-9. 39255850

[74] R. D. Teo , R. Wang , E. R. Smithwick , A. Migliore , M. J. Therien , D. N. Beratan , Proc. Natl. Acad. Sci. USA 2019, 116, 15811–15816, 10.1073/pnas.1906394116. 31341081 PMC6689991

[75] P. Nordlund , B.‐M. Sjöberg , H. Eklund , Nature 1990, 345, 593–598, 10.1038/345593a0. 2190093

[76] D. E. Westmoreland , P. R. Feliciano , G. Kang , C. Cuic , A. Kimc , J.‐A. Stubbe , D. G. Nocera , C. L. Drennan , Proc. Nat. Acad. Sci. 2024, 121, e2417157121, 10.1073/pnas.2417157121. 39475643 PMC11551348

[77] J. A. Cotruvo , J.‐A. Stubbe , Annu. Rev. Biochem. 2011, 80, 733–767, 10.1146/annurev-biochem-061408-095817. 21456967 PMC4703083

[78] M. Högbom , J. Biol. Inorg. Chem. 2010, 15, 339–349, 10.1007/s00775-009-0606-5. 20225400

[79] H. B. Gray , J. R. Winkler , Q. Rev. Biophys. 2003, 36, 341–372, 10.1017/S0033583503003913. 15029828

[80] M. Cordes , B. Giese , Chem. Soc. Rev. 2009, 38, 892, 10.1039/b805743p. 19421569

[81] B. Giese , M. Napp , O. Jacques , H. Boudebous , A. M. Taylor , J. Wirz , Angew. Chem. Int. Ed. Engl. 2005, 44, 4073–4075, 10.1002/anie.200500391. 15915526

[82] B. Giese , M. Wang , J. Gao , M. Stolz , P. Müller , M. Graber , J. Org. Chem. 2009, 74, 3621–3625, 10.1021/jo900375f. 19344128

[83] J. Gao , P. Müller , M. Wang , S. Eckhardt , M. Lauz , K. M. Fromm , B. Giese , Angew. Chem. Int. Ed. Engl. 2011, 50, 1926–1930, 10.1002/anie.201003389. 21328672

[84] E. Meggers , M. E. Michel‐Beyerle , B. Giese , J. Amer. Chem. Soc. 1998, 120, 12950–12955, 10.1021/ja983092p.

[85] M. Bixon , B. Giese , S. Wessely , T. Langenbacher , M. E. Michel‐Beyerle , J. Jortner , Proc. Natl. Acad. Sci. USA 1999, 96, 11713–11716, 10.1073/pnas.96.21.11713. 10518515 PMC18351

[86] B. Giese , M. Spichty , S. Wessely , Pure & Appl. Chem. 2001, 73, 449–453, 10.1351/pac200173030449.

[87] B. Giese , J. Amaudrut , A. K. Köhler , M. Spormann , S. Wessely , Nature 2001,412, 318–320, 10.1038/35085542. 11460159

[88] V. Chabert , L. Babel , M. P. Fueeg , M. Karamash , E. S. Madivoli , N. Herault , J. M. Dantas , C. A. Salgueiro , B. Giese , K. M. Fromm , Angew. Chem. Int. Ed. Engl. 2020, 59, 12331–12336, 10.1002/anie.201914873. 31815351

[89] M. Karamash , M. Stumpe , J. Dengjel , C. A. Salgueiro , B. Giese , K. M. Fromm , Front. Microbiol. 2022, 13, 909109, 10.3389/fmicb.2022.909109. 35783399 PMC9248073

[90] D. R. Lovley , T. Ueki , T. Zhang , N. S. Malvankar , P. M. Shrestha , K. A. Flanagan , M. Aklujkar , J. E. Butler , L. Giloteaux , A.‐E. Rotaru , D. E. Holmes , A. E. Franks , R. Orellana , C. Risso , K. P. Nevin , Adv. Microb. Physiol. 2011, 59, 1. 22114840 10.1016/B978-0-12-387661-4.00004-5

[91] G. Reguera , K. D. McCarthy , T. Mehta , J. S. Nicoll , M. T. Tuominen , D. R. Lovley , Nature 2005, 435, 1098–1101, 10.1038/nature03661. 15973408

[92] L. P. Nielsen , N. Risgaard‐Petersen , H. Fossing , P. B. Christensen , M. Sayama , Nature 2010, 463, 1071. 20182510 10.1038/nature08790

[93] M. Z. Tabari , A. L. Hochbaum , Front. Chem. 2025, 13, 1621274, 10.3389/fchem.2025.1621274. 40740311 PMC12307469

[94] D. L. Lovley , Front. Chem. 2025, 13, 1674350, 10.3389/fchem.2025.1674350. 41189673 PMC12580347

[95] C. Shen , A. I. Salazar‐Morales , W. Jung , S. E. Yalcin , F. A. Samatey , N. S. Malvankar , Cell Chem. Biol. 2025, 32, 239–254, 10.1016/j.chembiol.2024.12.013. 39818215 PMC11845295

[96] L. Digel , M. L. Justesen , N. S. Madsen , N. Fransaert , K. Wouters , R. Bonné , L. E. Plum‐Jensen , I. P. G. Marshall , P. B. Jensen , L. Nicalas‐Asselineau , T. Drace , A. Boeggild , J. L. Hansen , A. Schramm , E. D. Boejesen , L. P. Nielsen , J. V. Manca , T. Boesen , EMBO Rep. 2025, 26, 1749–1767, 10.1038/s44319-025-00387-8. 39962228 PMC11976967

[97] B. Giese , M. Karamash , K. M. Fromm , FEBS Lett. 2023, 597, 166–173, 10.1002/1873-3468.14493. 36114008

[98] F. J. R. Meysman , B. Smets , S. H. Martinez , N. Claes , B. C. Schroeder , J. S. Geelhoed , Y. Liu , J. Choyikutty , T. Chennit , T. Bodson , A. Collauto , M. M. Roessler , D. Karpov , S. Bohic , M. Aramini , S. Hayama , M. Wetherington , M. A. Zwijnenburg , G. Pankratova , I. Pintelon , J.‐P. Timmermans , G. Nuyts , K. DeWael , S. Bals , J. Verbeeck , H. Remaut , H. T. S. Boschker , bioRxiv 2025, 10.1101/2025.10.10.681601.

