# Supplementary material for: 50 Years of Giese Reaction – a Personal View
Source: Angew Chem Int Ed Engl. 2025 Dec 12;65(7):e24825. doi: 10.1002/anie.202524825 (PMC12887624; doi:10.1002/anie.202524825)
Supplement: Supplementary file 1 — Supporting Information [file ANIE-65-e24825-s001.docx]

**Supplemental Information**

**"50 Years of Giese Reaction"**

Martin Spichty, Hendrik Zipse, Salem Majouri, Katharina M. Fromm, Bernd Giese

**Kinetics Simulations**

**Introduction**

The Giese reaction is a synthetically useful C-C bond forming process and involves the addition of alkyl radicals to Michael acceptors, followed by a hydrogen atom transfer step to yield the final addition products. A representative example is shown in Scheme S1, where the reaction of cyclohexyl iodide (**1**, 1.0 eq.) with acrylonitrile (**2,** 5.0 eq.) yields 95% of addition product **3** in the presence of tri-*n*-butyltin hydride (**4**) as the H-donor under photochemical initiation conditions in ethanol at 25 ^o^C.^[1,2]^ Tri-*n*-butyltin hydride (**4**) is generated through reaction of sodium borohydride (NaBH_4_ (**5**), 1.3 eq.) with either the small amount (0.2 eq.) of tri-*n*-butyltin chloride (**6**) added at the beginning of the reaction, or with tri-*n*-butyltin iodide (**7**) formed through reaction of iodide **1** with tin-based radicals. Based on the reaction description, the reaction appears to be complete after approx. 2 - 4 h. The remarkably high yield of 95% of **3** implies that direct reduction of iodide **1** to cyclohexane (**8**) as the most likely side product represents not more than 5% of the starting iodide **1**. Unfortunately, the degree to which this side reaction actually occurs has not been reported for this example.

**Scheme S1**. Giese reaction of cyclohexyl iodide (**1**) with acrylonitrile (**2**).

A reaction mechanism accounting for all observables of this transformation is shown in Scheme S2. The reaction is initiated by photolytic cleavage of the C-I bond in iodide **1** (reaction (1)) to yield the chain carrying cyclohexyl radical **9** and atomic iodine (**10**). Most of iodide **1** is, however, consumed in the actual chain process and not in the photolytic initiation step. Assuming that the chain is three to four orders of magnitude more efficient in turning over iodide **1** as compared to the initiation step, we select *k*_1_ = 2.0E-08 s^-1^ as an "effective" first-order rate constant for the initiation process. The rate of this process also depends on the concentration of iodide **1**, whose initial value is set to [**1**]_0_ = 0.1 M based on the available reaction description. The initial concentrations of all other reactants then equate to [**2**]_0_ = 0.5 M, [**6**]_0_ = 0.02 M, and [**5**]_0_ = 0.13 M. While cyclohexyl radical **9** feeds directly into the productive chain, iodine radical **10** most likely reacts with tin hy dride **4** in a second chain-initiating event to yield tin radical **11** and HI (**12**). The rate of this transformation is not known but is assumed to be quite fast. This is also true for the reaction of the HI (**12**) acid formed in its reaction with NaBH_4_ (**5**).

**Scheme S2**. Reaction mechanism for the Giese reaction shown in Scheme S1.

The first of the three chain-carrying steps involves addition of radical **9** to acrylonitrile (**2**) (eq. 3), which has been suggested to proceed with *k*_3_ = 1.0E+06 M^-1^ s^-1^.^[2]^ This value appears best suited for secondary alkyl radicals considering the data available for different radicals under a variety of conditions. Fischer and coworkers have reported substantial solvent polarity effects for the reaction of *tert*-butyl radicals to acrylonitrile at 300 K, with rate constants of *k*(tBu) = 5.4E+06 M^-1^ s^-1^ in isopropanol, and 2.8E+06 M^-1^ s^-1^ in tetradecane.^[6]^ Similarly, Minisci and coworkers have reported *k*(iPr) = 4.3E+06 M^-1^ s^-1^ for the reaction of isopropyl radicals with acrylonitrile at 273.15 K in acetonitrile.^[9]^ At the low end of rate constants we find the value of *k*(prim) = 5.4E+05 M^-1^ s^-1^ determined by Giese et al. for addition of the primary 5-hexenyl radical to acrylonitrile at 293.15 K in dichloromethane.^[12]^ In the second chain-carrying step adduct radical **13** reacts with tin hydride **4** to give tin radical **11** and product **3** (eq. 4). The exact kinetics of this step have not been determined, but laser flash photolysis (LFP) measurements for reactions of hydride **4** with an array of alkyl radicals (ethyl, n-butyl, isopropyl, cyclohexyl, *tert*-butyl) indicate largely similar rate constants of *k*_4_ = 2.0E+06 M^-1^ s^-1^ in isooctane solution at 300 K (25 ^o^C), and we may thus adopt this value also for the reaction studied here. The third chain-carrying step involves iodine abstraction from iodide **1** by tin radical **11** to give tin iodide **7** and cyclohexyl radical **9** (eq. 5). Reactions of tin radical **11** with alkyl iodides are known to be very fast, often reaching values beyond 1.0E+09 M^-1^ s^-1^ at 300 K. Exact values are difficult to determine due to technical challenges, and we may thus adopt *k*_5_ = 1.0E+09 M^-1^ s^-1^ as the lower limit for this reaction step.^[7,8]^

Reaction of cyclohexyl radical **9** with tin hydride **4** is a notable side reaction under many synthetically important conditions, which can be minimized through administration of low tin hydride concentrations. The rate constant for alkyl radicals is *k*_4_ = 2.0E+06 M^-1^ s^-1^. The second side reaction involves addition of adduct radical **13** to a second acrylonitrile monomer **2** to generate the double adduct **P**. The rate constant for this addition step is expected to be substantially lower compared to addition step (3) due to polar effects and we assume here an upper limit of *k*_7a_ = 1.0E+03 M^-1^ s^-1^ for this step.^[10]^ Further reactions of double adduct **P** may involve continued addition to acrylonitrile (**2**) and thus the formation of oligomeric acrylonitrile species, or trapping by hydride (**4**). The latter is assumed here as the more relevant (faster) process with *k*_7b_ = 2.0E+06 M^-1^ s^-1^.

All chain carrying steps will compete with chain-terminating recombination between any of the chain-carrying radicals. The rate constants of these recombination reactions are not known individually, and we thus assume an average value of *k*_8_ = 5.0E+09 M^-1^ s^-1^ at 300 K based on diffusive encounter statistics in organic solvents.^[5]^ The off-cycle (that is, non-radical) reaction of tin halides with NaBH_4_ appears to be overall faster than the radical chain-mediated formation of tin iodide **7**.^[5]^ We may thus safely assume that, already on mixing the initial reaction solution, all of tin chloride **6** is reduced to tin hydride **4** by sodium borohydride **5**. At the onset of the photolytic activation of iodide **1**, the initial concentrations thus are [**4**]_0_ = 0.02 M and [**5**]_0_ = 0.11 M. The rate constant *k*_8_ for the subsequent reduction of tin iodide **7** was then selected such that the concentration of tin hydride (**4**) approaches that of the initially added tin chloride of [**6**]_0_ = 0.02 M at all stages of the reaction. The rate constant for the reaction of NaBH_4_ with HI (**12**) is not known but assumed to be as fast as reaction of NaBH_4_ with iodide **7** for the sake of simplicity.

**Kinetics Simulations**

The photolytic activation of alkyl iodides is commonly assumed to follow a first-order rate law under the condition of a constant flux of photons,^[13]^ and no additional factors limiting light absorption of iodide substrates. The consumption of iodide **1** under continous photolysis conditions can then be described by rate equation (1a):

-d[**1**]/d*t* = *k*_1_[**1**] (1a)

[**1**] = [**1**]_0_ exp(-*k_1_*t) (1b)

ln[**1**] = ln[**1**]_0_ - *k_1_*t (1c)

*t_1/2_* = ln(2)/*k*_1_ (1d)

where the effective rate constant *k*_1_ includes all factors associated with the flux and the absorption of incoming photons. The time course of the concentration of iodide **1** can then be traced by two different methods. The first relies on the integrated solution of differential equation (1a), which is either used in its exponential form (1b) or its logarithmic form (1c). The reaction time *t* (in s) commonly refers to the time since the onset of photolysis, the initial substrate concentration being [**1**]_0_ = 0.1 M and the effective rate constant being *k*_1_ = 2.0E-08 s^-1^. The reaction half-life *t_1/2_* as given by eq. (1d), where 50% of iodide **1** is consumed, is actually quite long at *t_1/2_* = 3.47E+07 s (or 401 days). A pictorial representation of the turnover of **1** as described by the integrated rate law (1b) at ten representative reaction times is shown in Figure S1.


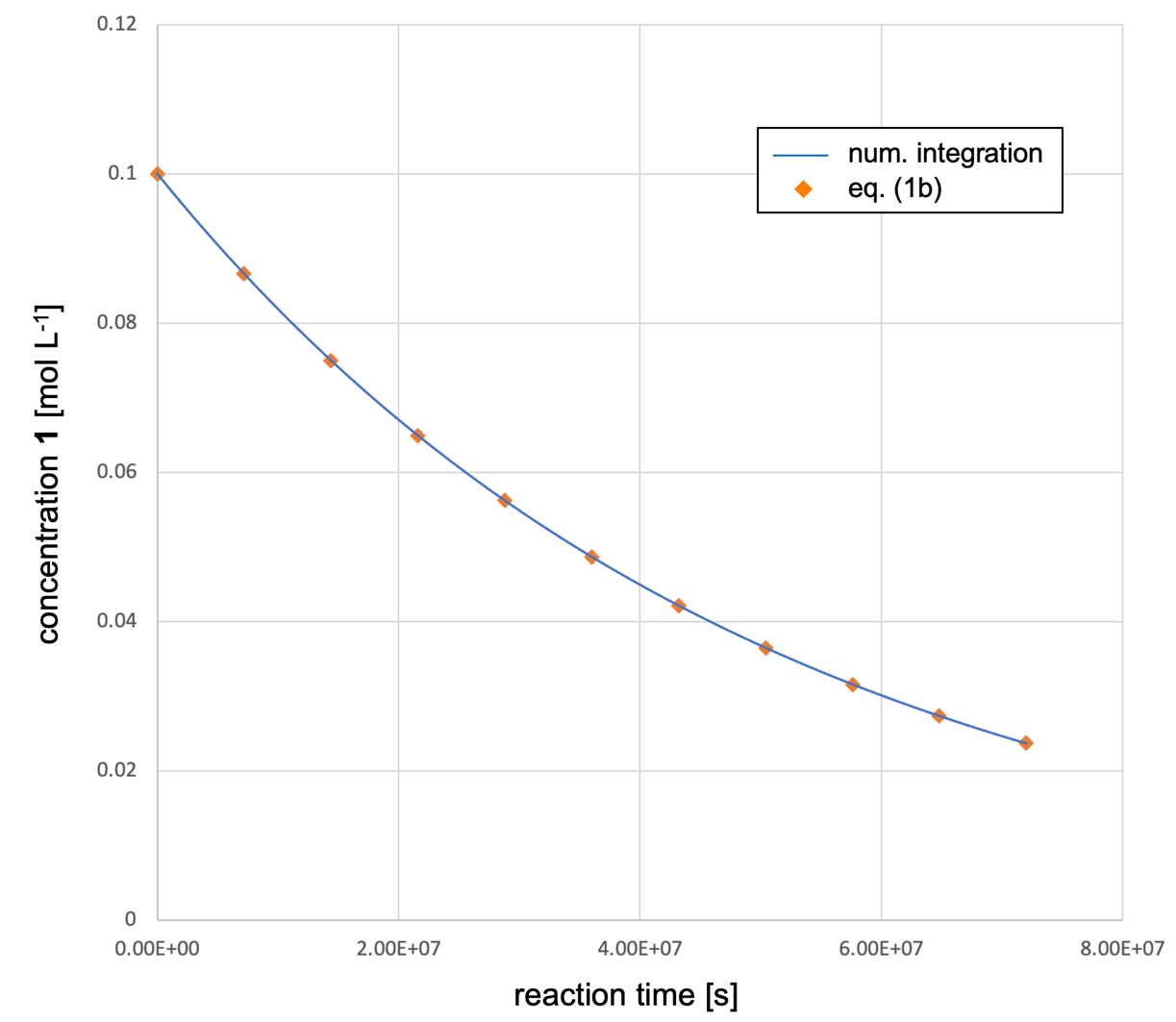


**Figure S1**. Time dependence of photolytic activation of iodide **1**.

While the use of integrated rate laws is highly practical for individual reaction steps, this is not so for the multistep reaction mechanism outlined in Scheme S2, where the rate laws of individual reaction steps combine into a larger set of ordinary differential equations (ODEs). An important characteristic of the rate equations for radical chain chemistry is that a set of comparatively slow reactions (such as initiation and off-cycle reactions) combine with comparatively fast reactions (chain and termination steps) into one set of ODEs. These can be solved numerically with robust algorithms such as the LSODA solver implemented in the microkinetics simulation program COPASI.^[11]^ After selecting an appropriate time (integration) step for the system at hand, the ODE solver moves forward in time in a stepwise manner while continously updating the concentrations of all species. For the photolytic activation of iodide **1**, the time course of [**1**] has been predicted in this way selecting a comparatively long time step of Δ*t* = 72000 s (which corresponds to 1000 steps for the blue turnover curve shown in Figure S1). At the end of the simulation shown in Figure S1, the iodide concentration has dropped from 0.1 M to [**1**] = 0.0237 M in *t* = 7.20E+07 s, which equates to (0.1 - 0.0237)/0.1 = 76.3% turnover. For the combined reaction system shown in Scheme S2 significantly shorter time steps (Δ*t* = 0.01 s for the initial 3 s of the reaction time, Δ*t* = 0.25 s for complete tunrover curves) have been used. In all cases it has been verified that the choice of step size has no notable impact on the simulation outcome.

**Simulation I - basic characteristics of radical chain chemistry**

Simulation I focuses on basic characteristics of the proposed substrate mechanism and includes the following rate equations:

1) Initiation

-d[**1**]/d*t* = *k*_1_[**1**] (1)

-d[**10**]/dt = *k*_2_[**10**][**4**] (2)

2) 3-Step chain

d[**13**]/dt = *k*_3_[**9**][**2**] (3)

d[**3**]/dt = *k*_4_[**13**][**4**] (4)

d[**7**]/dt = *k*_5_[**11**][**1**] (5)

3) Termination

The rate constants for radical recombination reactions are assumed to be identical for all radicals generated in the initiation or the chain steps. Dimerization of iodine radical **10** is not assumed to be relevant simply due to the instability of the product formed.

d[**9x9**]/dt = *k*_8_[**9**][**9**] (8a)

d[**11x11**]/dt = *k*_8_[**11**][**11**] (8b)

d[**9x11**]/dt = *k*_8_[**9**][**11**] (8c)

d[**9x13**]/dt = *k*_8_[**9**][**13**] (8d)

d[**11x13**]/dt = *k*_8_[**11**][**13**] (8e)

d[**13x13**]/dt = *k*_8_[**13**][**13**] (8f)

d[**9x10**]/dt = *k*_8_[**9**][**10**] (8g)

d[**10x11**]/dt = *k*_8_[**10**][**11**] (8h)

d[**10x13**]/dt = *k*_8_[**10**][**13**] (8i)

4) Off-cycle reactions

The rates for formation of tin hydride **4** from iodide **7** and for the formation of H_2_ (**15**) from HI (**12**) are assumed to be identical:

d[**4**]/d*t* = *k*_9_[**5**][**7**] (9)

d[**15**]/d*t* = *k*_10_[**5**][**12**] (10)

Initial concentrations are:

[**1**]_0_ = 0.1 M

[**2**]_0_ = 0.5 M

[**4**]_0_ = 0.02 M

[**5**]_0_ = 0.11 M

Rate constants are:

*k*_1_= 2.0E-08 s^-1^

*k*_2_ = 5.0E+09 M^-1^ s^-1^

*k*_3_ = 1.0E+06 M^-1^ s^-1^

*k*_4_ = 2.0E+06 M^-1^ s^-1^

*k*_5_ = 1.0E+09 M^-1^ s^-1^

*k*_8_ = 5.0E+09 M^-1^ s^-1^

*k*_9_ = 1.0E+00 M^-1^ s^-1^

*k*_10_ = 1.0E+00 M^-1^ s^-1^

In **Simulation I** we neglect all side reactions of the radical chain process. The first snapshot of the reaction shown in Figure S2 illustrates that a productive radical chain for this reaction is established within 1.0 s after starting the photolytic activation of iodide **1**. The three chain carrying radicals have rather different concentrations at this point with [**9**] = 4.8E-11 M, [**13**] = 6.1E-10 M, and [**11**] = 2.4E-13 M. As already mentioned before, these concentrations result from the numerical microkinetics simulations with a time step of Δ*t* = 0.01 s. The concentration values then combine with rate constants and the rate equations described above to define the actual reaction rates for each reaction step. The differences in radical concentrations are the result of the requirement that the actual reaction rates of all steps of an established chain process have to be (largely) identical.^[5]^ For the current example, the actual rates r(x) (or flux values) of the chain steps are:

r(3) = *k*_3_[**9**][**2**] = 1.0E+06 M^-1^ s^-1^ x 4.8E-11 M x 0.5 M = 2.4E-05 mol s^-1^

r(4) = *k*_4_[**13**][**4**] = 2.0E+06 M^-1^ s^-1^ x 6.1E-10 M x 0.02 M = 2.4E-05 mol s^-1^

r(5) = *k*_5_[**11**][**1**] = 1.0E+09 M^-1^ s^-1^ x 2.4E-13 M x 0.1 M = 2.4E-05 mol s^-1^


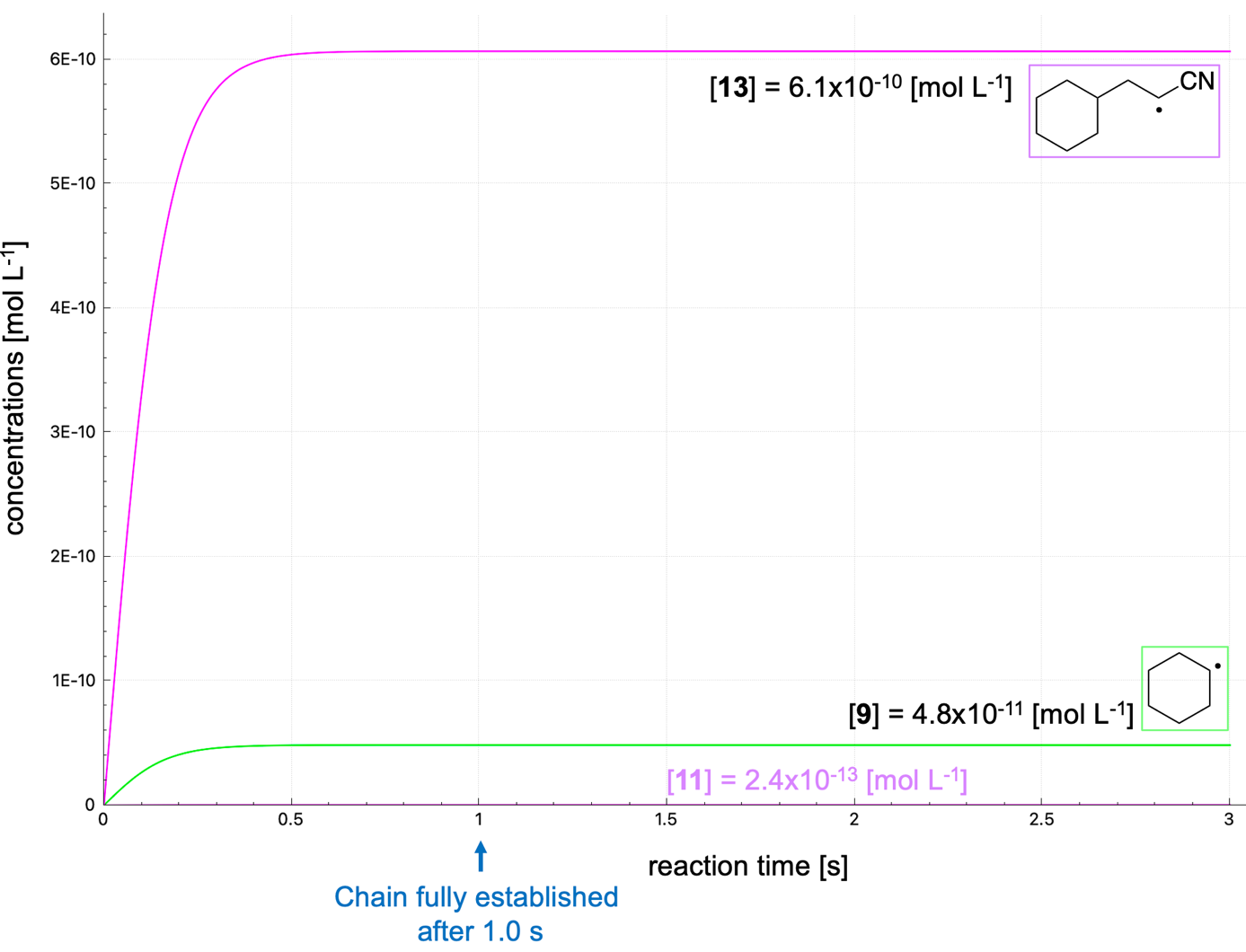


**Figure S2**. Radical concentrations at the beginning of the Giese reaction (*t* = 1 s) shown in Scheme S1.

At the beginning of the reaction (at *t* = 1 s) the three rate values of the chain carrying steps amount to 2.4±0.1E-05 mol s^-1^, where the rate of initiation is comparatively high due to the high concentration of starting iodide **1**. Minor numerical differences between the three values reflect the influence of the initiating and terminating steps. The concentration of tin radical **11** is much lower (2.4E-13 M) than that of cyclohexyl radical **9** (4.8E-11 M) due to the high rate constant for its reaction with iodide **1**. The concentration of adduct radical **13** is highest (at 6.1E-10 M) due to the comparatively low concentration of its reaction partner (Bu_3_SnH) in chain step (4).

How productive the current chain process is as compared to the termination steps can best be seen by comparing the rate of the product forming step (r(4) = 2.4E-05 mol s^-1^) with the rates of the termination steps. The most dominant of the terminating steps involves the dimerization of radical **13** due to its comparatively high concentration. At *t* = 1 s the rate of this process amounts to r(8f) = *k*_8_[**13**][**13**] = 5.0E+09 M^-1^ s^-1^ x 6.1E-10 M x 6.1E-10 M = 1.9E-9 mol s^-1^. From the ratio of these two steps of r(4)/r(8f) = 12900 we see that the chain turns over more than 10000 times before being terminated through radical recombination.

A second snapshot of this reaction (Figure S3) is taken after the first half life (at *t* = 2450 s) when 50% of starting iodide **1** is consumed. The concentrations of the chain-carrying radicals are slightly lower at this point for cyclohexyl radical **9** (3.7E-11 M) and adduct radical **13** (4.3E-10 M), but slightly higher for tin radical **11** (3.4E-13 M). With these changes the radical concentrations respond to two factors: (a) the reduced rate of initiation through reduction of the concentration of iodide **1**; and (b) the change in reactant concentrations. The latter factor is relevant because it directly impacts the requirement that the reaction rates of all three chain steps have to be identical (at 1.7E-05 mol s^-1^), despite the changes in reactant concentrations:

r(3) = *k*_3_[**9**][**2**] = 1.0E+06 M^-1^ s^-1^ x 3.7E-11 M x 0.45 M = 1.7E-05 mol s^-1^

r(4) = *k*_4_[**13**][**4**] = 2.0E+06 M^-1^ s^-1^ x 4.3E-10 M x 0.02 M = 1.7E-05 mol s^-1^

r(5) = *k*_5_[**11**][**1**] = 1.0E+09 M^-1^ s^-1^ x 3.4E-13 M x 0.05 M = 1.7E-05 mol s^-1^


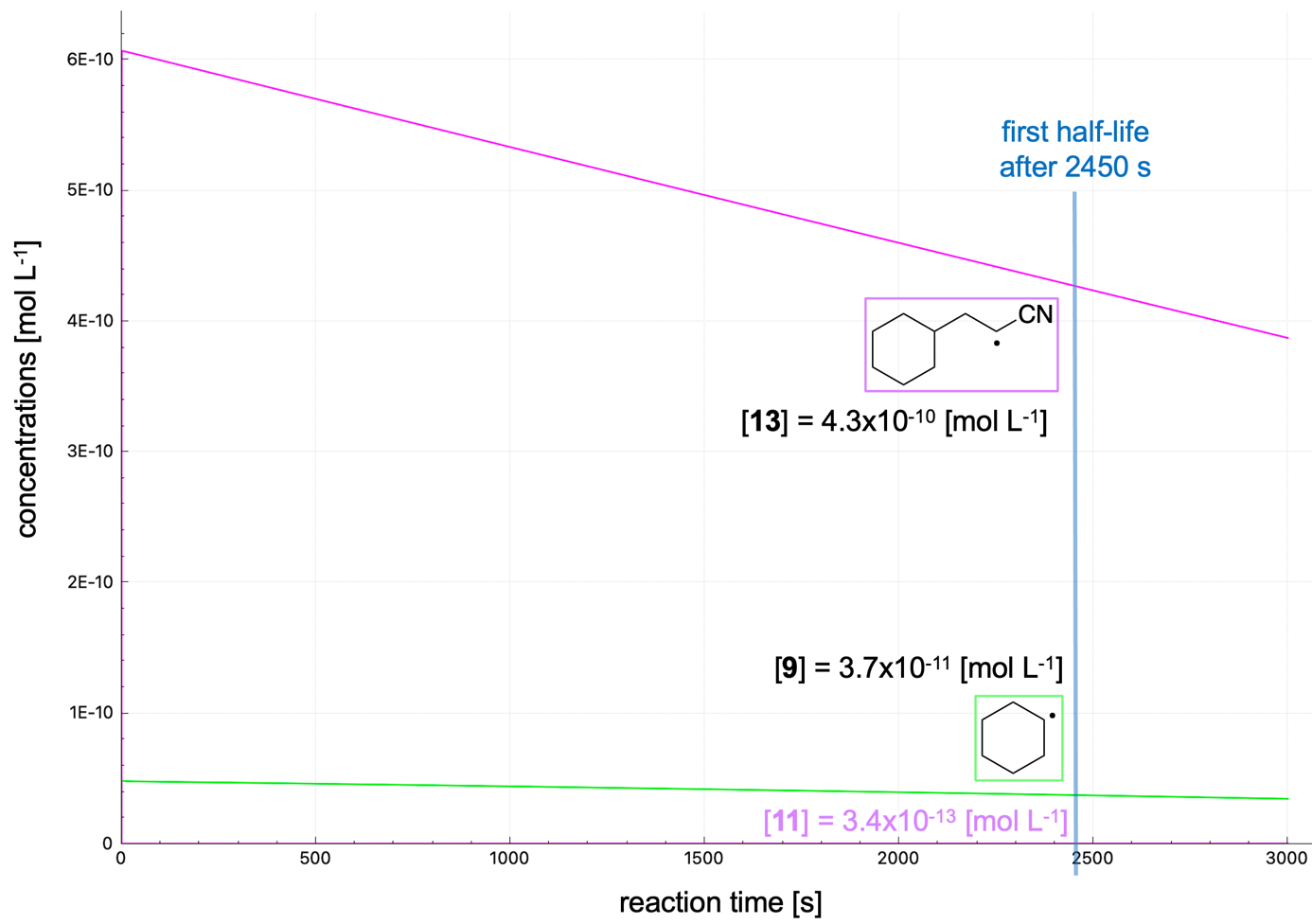


**Figure S3**. Radical concentrations after the first half-life (*t* = 2450 s) of the Giese reaction shown in Scheme S1.

The most dominant of the terminating steps still involves the dimerization of radical **13** and amounts to r(8f) = *k*_8_[**13**][**13**] = 5.0E+09 M^-1^ s^-1^ x 4.3E-10 M x 4.3E-10 M = 9.2E-10 mol s^-1^. From the ratio of these two steps of r(4)/r(8f) = 18480 we see that the chain efficiency is still very high.

A third snapshot of this reaction (shown in Figure S4) is taken after a reaction time of 7200 s (2 h), where turnover of iodide **1** amounts to 98%. The concentrations of the chain-carrying radicals are lower at this point for cyclohexyl radical **9** (8.48E-12 M) and adduct radical **13** (8.65E-11 M), and again higher for tin radical **11** (1.62E-12 M). The rates of the three chain steps continue to be comparable at this point (at 3.4E-06 mol s^-1^):

r(3) = *k*_3_[**9**][**2**] = 1.0E+06 M^-1^ s^-1^ x 8.48E-12 M x 0.40 M = 3.4E-06 mol s^-1^

r(4) = *k*_4_[**13**][**4**] = 2.0E+06 M^-1^ s^-1^ x 8.65E-11 M x 0.0 M = 3.4E-06 mol s^-1^

r(5) = *k*_5_[**11**][**1**] = 1.0E+09 M^-1^ s^-1^ x 1.62E-12 x 0.0021 M = 3.4E-06 mol s^-1^

and the ratio of the chain flux relative to recombination of radical **13** as a reflection of chain length remains large at r(4)/r(8f) = 90880. This impressively high value not only points to the productive nature of the chain process under study here, but also explains why the rather low (absolute) rate of initiation documented in Figure S1 can drive the reaction to completion in approx. 2 h of reaction time.


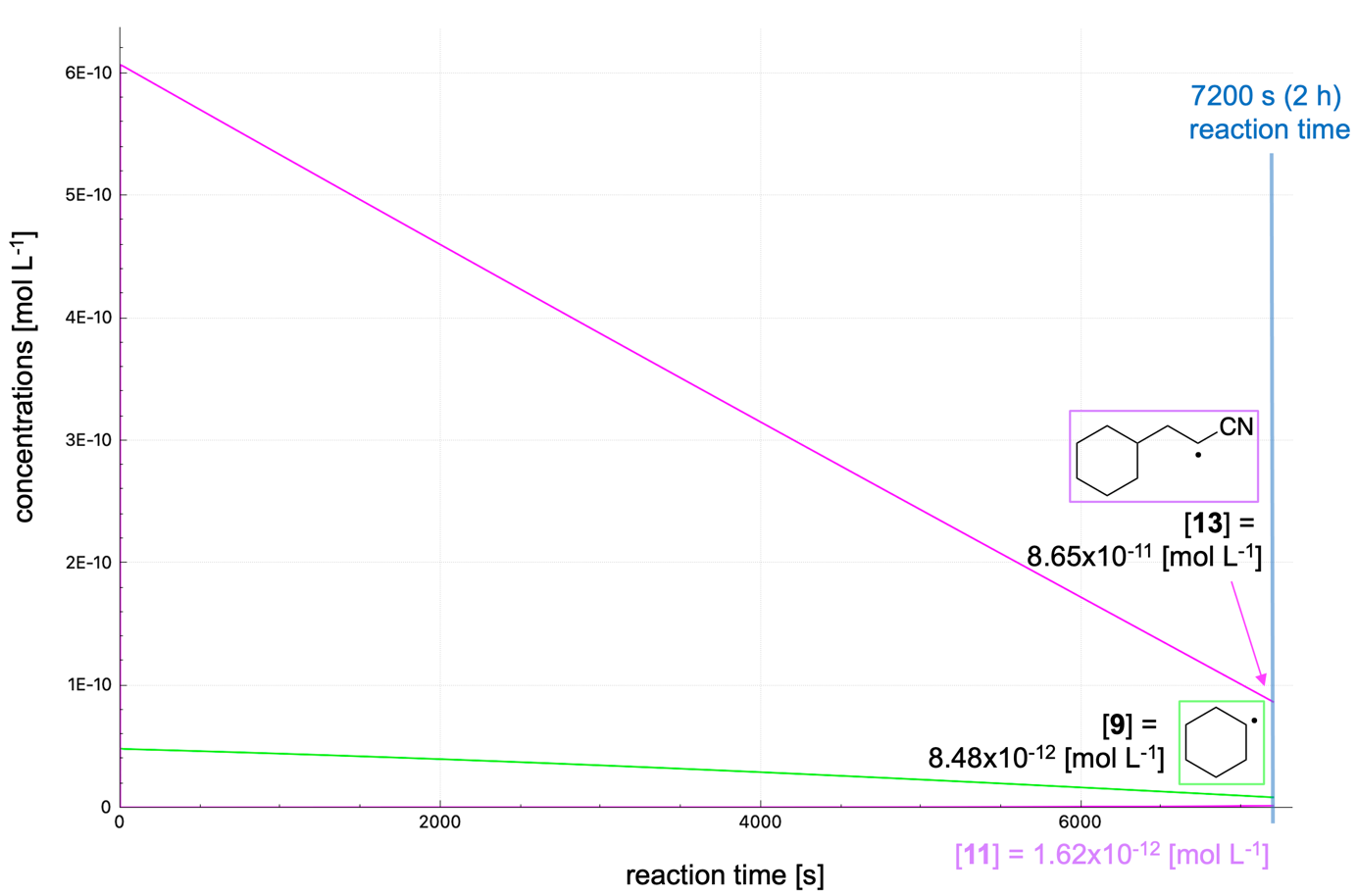


**Figure S4a**. Radical concentrations after a reaction time of 7200 s (2 h) in the Giese reaction shown in Scheme S1.

The concentrations of cyclohexyl iodide (**1**), reductant NaBH_4_ (**5**), and adduct **3** shown in Figure S4b follow the expected exponential behaviour, while the concentration of HSnBu_3_ (**4**) remains constant over time at [**4**] = 0.02 M. This latter observation confirms that the off-cycle rate of reduction of tin iodide **7** with NaBH_4_ (**5**) is always faster than formation of **7** in the radical chain process.


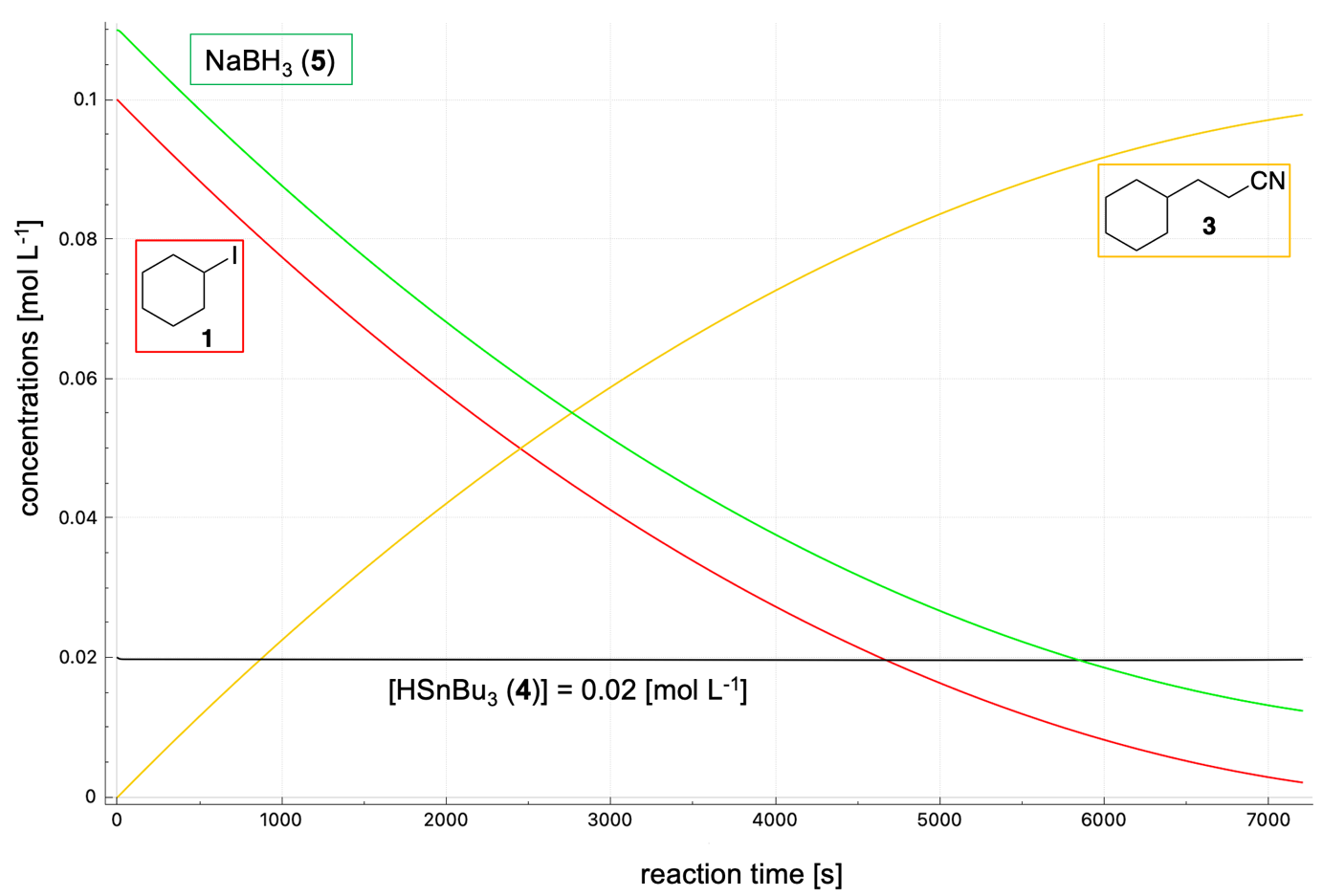


**Figure S4b**. Selected substrate and product concentrations within a reaction time of 7200 s (2 h) in the Giese reaction shown in Scheme S1.

In summary we can conclude from the results of Simulation 1 that the radical concentrations in an established chain process are neither constant nor identical but respond to (a) the variable rate of chain initiation (which decreases over time as a consequence of the decrease of iodide concentration [**1**]); and (b) the change in reactant concentrations. More importantly, radical and substrate concentrations respond to the requirement of identical reaction rates for all steps of an established chain process at all stages of the reaction studied here (that is, more or less independent of the degree of turnover).

**Simulation II - the influence of competing chain processes**

Simulation II combines all steps of Simulation I and adds the effects of competing side reactions of the chain steps. The actual rate equations used for these reactions are:

5) Side reactions

d[**8**]/dt = *k*_6_[**9**][**4**] (6)

d[**P**]/dt = *k*_7a_[**13**][**2**] (7a)

d[**PH**]/dt = *k*_7b_[**P**][**4**] (7b)

with rate constants:

*k*_6_ = 2.0E+06 M^-1^ s^-1^

*k*_7a_ = 1.0E+03 M^-1^ s^-1^

*k*_7b_ = 2.0E+06 M^-1^ s^-1^

Side reaction (7a) also adds radical **P** as a new transient species, which will participate in chain termination events through recombination reactions. The list of rate equations for chain termination thus includes the following additional entries, all with the same recombination rate constant *k*_8_ = 5.0E+09 M^-1^ s^-1^:

d[**9xP**]/dt = *k*_8_[**9**][**P**] (8j)

d[**10xP**]/dt = *k*_8_[**10**][**P**] (8k)

d[**11xP**]/dt = *k*_8_[**11**][**P**] (8l)

d[**13xP**]/dt = *k*_8_[**13**][**P**] (8m)

d[**PxP**]/dt = *k*_8_[**P**][**P**] (8n)

The first snapshot of Simulation II in Figure S5 illustrates that a productive radical chain for this reaction is again established within 1.0 s after starting the photolytic activation of iodide **1**. The three chain carrying radicals have concentrations of [**9**] = 4.91E-11 M, [**13**] = 6.06E-10 M, and [**11**] = 2.65E-13 M and are thus hardly altered through the presence of the two side reactions. The reaction rates r(x) of the chain steps are:

r(3) = *k*_3_[**9**][**2**] = 1.0E+06 M^-1^ s^-1^ x 4.91E-11 M x 0.5 M = 2.45E-05 mol s^-1^

r(4) = *k*_4_[**13**][**4**] = 2.0E+06 M^-1^ s^-1^ x 6.06E-10 M x 0.02 M = 2.42E-05 mol s^-1^

r(5) = *k*_5_[**11**][**1**] = 1.0E+09 M^-1^ s^-1^ x 2.65E-13 M x 0.1 M = 2.64E-05 mol s^-1^

Numerically, these values are closely similar to those seen after *t* = 1 s already in Simulation I in the absence of side reactions, but the differences between the three chain steps are now larger as a consequence of two additional side-reaction chains competing for the same transient radicals. From the product concentrations predicted after *t* = 1s of [**3**] = 2.1E-05 M, [**8**] = 1.7E-06 M and [**PH**] = 2.6E-07 M we see that cyclohexane formation in side reaction (6) is more competitive with [**3**]/[**8**] = 12.4 as compared to oligomer formation in side reaction (7a)/(7b) with [**3**]/[**PH**] = 80.8.


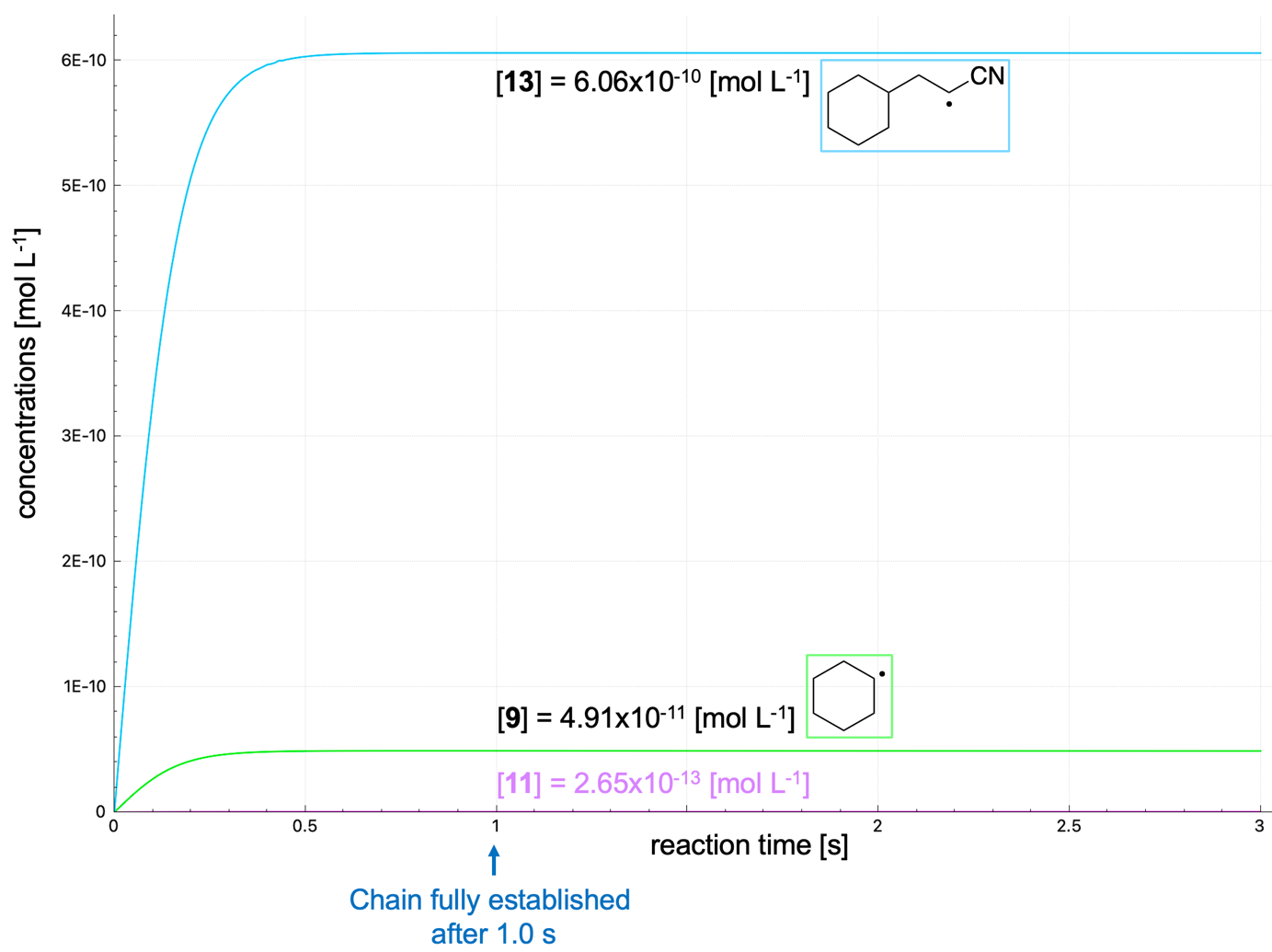


**Figure S5**. Radical concentrations after a reaction time of 1 s in the Giese reaction shown in Scheme S1 (including side reactions).

When running Simulation II to the same extended reaction time of t = 7200 s (2 h) as in Simulation I, we obtain radical concentrations for cyclohexyl radical **9** (3.71E-12 M) and adduct radical **13** (3.78E-11 M), and again higher for tin radical **11** (3.77E-12 M).

The rates of the three chain steps continue to be similar at this point:

r(3) = *k*_3_[**9**][**2**] = 1.0E+06 M^-1^ s^-1^ x 3.71E-12 M x 0.407 M = 1.51E-06 mol s^-1^

r(4) = *k*_4_[**13**][**4**] = 2.0E+06 M^-1^ s^-1^ x 3.78E-11 M x 0.02 M = 1.51E-06 mol s^-1^

r(5) = *k*_5_[**11**][**1**] = 1.0E+09 M^-1^ s^-1^ x 3.77E-12 M x 0.00044 M = 1.66E-06 mol s^-1^

but we again note the impact of the additional side reaction chains. Comparing the rate of product formation r(4) = 1.51E-06 mol s^-1^ with that of termination through recombination of radical **13** r(8f) = *k*_8_[**13**][**13**] = 5.0E+09 M^-1^ s^-1^ x 3.78E-11 M x 3.78E-11 M = 7.14E-12 mol s^-1^ we see that the chain is very effective with r(4)/r(8f) = 211360. From the final concentrations of reactants and products of [**1**] = 0.00044 M, [**2**] = 0.407 M, [**3**] = 0.0905 M, [**8**] = 0.00798 M and [**PH**] = 0.00104 M we see that at essentially complete turnover of iodide **1** (99.6 %) the product ratio between addition product **3** and cyclohexane (**8**) amounts to 0.0905 M/0.00798 M = 11.34. Under common workup conditions, cyclohexane (**8**) will be removed from the reaction mixture together with all solvents. Based on the above concentrations, an isolated yield for adduct **3** of 0.0905/0.1*100 = 90.5% is expected, which is somewhat lower than the experimentally reported yield of 95%. Considering the approximate nature of all rate constant assumptions made in the current simulations, this is as good as we can expect the agreement to be.

**Simulation III - the role of radical addition rates to alkenes**

Surveying the rate constant information for the addition of alkyl radicals to acrylonitrile, we had noted before that the value of *k*_3_ = 1.0E+06 M^-1^ s^-1^ used in Simulations I and II^[2]^ may actually be somewhat higher (possibly up to 5.0E+06 M^-1^ s^-1^).^[6,9]^ Leaving all other data (rate equations, starting concentrations and rate constants) unchanged, Simulation II was therefore repeated up to a reaction time of t = 7200 s (2 h) with *k*_3_ values between 1.0E+06 M^-1^ s^-1^ and 5.0E+06 M^-1^ s^-1^ in order to quantify its effect on the product distribution.

**Table S1**. Dependence of turnover of iodide **1** and yield of adduct **3** as a function of addition rate constant *k*_3_ after a reaction time of *t* = 7200 s (2 h).

| *k*_3_  (M^-1^ s^-1^) | [**1**]  (M) | turnover **1**  (%) | [**3**]  (M) | [**8**]  (M) | [**3**]/[**8**] | yield **3**  (%) |
| --- | --- | --- | --- | --- | --- | --- |
| 1.0E+06 | 0.00044 | 99.6 | 0.0905 | 0.00798 | 11.34 | 90.5 |
| **2.0E+06** | **0.00068** | **99.3** | **0.0941** | **0.00416** | **22.62** | **94.1** |
| 3.0E+06 | 0.00077 | 99.2 | 0.0953 | 0.00282 | 33.79 | 95.3 |
| 4.0E+06 | 0.00083 | 99.2 | 0.0959 | 0.00213 | 45.02 | 95.9 |
| 5.0E+06 | 0.00086 | 99.1 | 0.0963 | 0.00171 | 56.32 | 96.3 |

From the results collected in Table S1 we can see that the turnover of iodide **1** reaches beyond 99% for all *k*_3_ values considered here, but the product distribution between adduct **3** and cyclohexane **8** is significantly altered in that higher addition rate constants *k*_3_ translate into higher yields of adduct **3**. Adding the fact that the turnover of iodide **1** shows small differences for the simulations reported in Table S1, the simulation with *k*_3_ = 2.0E+06 M^-1^ s^-1^ seems closest to the reported experimental outcome. This is easily understood on the basis of the competing addition/reduction reaction pathways of cyclohexyl radical **9** shown in Scheme S3:

**Scheme S3**. Competing reaction pathways for cyclohexyl radical **9**.

Cyclohexyl radical **9** can either add to acrylonitrile **2** with rate constant *k*_3_ or react with tin hydride **4** with rate constant *k*_6_. From the rate expressions for these two pathways, we calculate the branching ratio r(3)/r(6) as a function of the reactant concentrations [**2**] and [**4**], and the rate constant *k*_3_ and *k*_6_. Leaving all other settings unchanged, it is immediately seen that any increase in addition rate constant *k*_3_ leads to an increase in adduct radical **13** and (after reductive trapping) addition product **3**. We note in passing that the rate of trapping adduct radical **13** with HSnBu_3_ may also be larger than assumed here due to polar effects. What the microkinetics simulations add to this simple analysis is the insight, that the selection made at the branching point shown in Scheme S3 indeed translates into the expected closed-shell product distribution. Comparing the yields predicted for selected *k*_3_ values in Table S1 with that reported experimentally (95 %) indeed supports the hypothesis, that the correct value of this rate constant under the experimental conditions used is 2.0E+06 M^-1^ s^-1^ rather than 1.0E+06 M^-1^ s^-1^ (used in the initial Simulations I and II).

**Simulation IV - does the rate of initiation matter?**

The "effective" first order rate constant of *k*_1_ = 2.0E-08 s^-1^ for the photochemical activation of iodide substrate **1** was selected on the basis of providing sufficient initiating power for the overall reaction to complete in 2-4 h. That the comparatively slow initiation reaction shown in Figure S1 is sufficient for the chain process under study here is due to the superb chain length of the product-forming chain reaction. What factors (e.g. the photon flux passing through the sample at a given wavelength, the absorption efficiency of **1** at a given wavelength, the excited state lifetimes and the cleavage efficiencies of the most relevant excited states) actually impact the underlying photophysics of this process will not be scrutinized here, but we can nevertheless explore the impact of variations in *k*_1_ on essential features of the overall chain process. To this end Simulation IV will repeat Simulation III with *k*_1_ values ranging from 1.0E-08 s^-1^ to 1.0E-07 s^-1^. The characteristics monitored will include: (a) the product distribution of adduct **3** and cyclohexane **8** after 99% conversion of iodide **1** and, after workup, the yield of addition product **3**; (b) the radical concentrations after a reaction time of *t* = 1 s; (c) the radical concentrations at 50% conversion of iodide **1**; (d) the chain length (CL) calculated as the ratio of the product forming step r(4) and the most prominent termination step r(8f) at 50% conversion of iodide **1**. That the above reaction characteristics will be slightly different than those described in Simulation III is due tot he fact that all data are measured at a specific turnover point (99%) rather than a constant reaction time. The rate equations, initial reactant concentrations, and rate constants chosen are those used in Simulation III:

1) Initiation

-d[**1**]/d*t* = *k*_1_[**1**] (1)

-d[**10**]/dt = *k*_2_[**10**][**4**] (2)

2) 3-Step chain

d[**13**]/dt = *k*_3_[**9**][**2**] (3)

d[**3**]/dt = *k*_4_[**13**][**4**] (4)

d[**7**]/dt = *k*_5_[**11**][**1**] (5)

3) Termination

d[**9x9**]/dt = *k*_8_[**9**][**9**] (8a)

d[**11x11**]/dt = *k*_8_[**11**][**11**] (8b)

d[**9x11**]/dt = *k*_8_[**9**][**11**] (8c)

d[**9x13**]/dt = *k*_8_[**9**][**13**] (8d)

d[**11x13**]/dt = *k*_8_[**11**][**13**] (8e)

d[**13x13**]/dt = *k*_8_[**13**][**13**] (8f)

d[**9x10**]/dt = *k*_8_[**9**][**10**] (8g)

d[**10x11**]/dt = *k*_8_[**10**][**11**] (8h)

d[**10x13**]/dt = *k*_8_[**10**][**13**] (8i)

d[**9xP**]/dt = *k*_8_[**9**][**P**] (8j)

d[**10xP**]/dt = *k*_8_[**10**][**P**] (8k)

d[**11xP**]/dt = *k*_8_[**11**][**P**] (8l)

d[**13xP**]/dt = *k*_8_[**13**][**P**] (8m)

d[**PxP**]/dt = *k*_8_[**P**][**P**] (8n)

4) Off-cycle reactions

d[**4**]/d*t* = *k*_9_[**5**][**7**] (9)

d[**15**]/d*t* = *k*_10_[**5**][**12**] (10)

5) Side reactions

d[**8**]/dt = *k*_6_[**9**][**4**] (6)

d[**P**]/dt = *k*_7a_[**13**][**2**] (7a)

d[**PH**]/dt = *k*_7b_[**P**][**4**] (7b)

Initial concentrations are:

[**1**]_0_ = 0.1 M

[**2**]_0_ = 0.5 M

[**4**]_0_ = 0.02 M

[**5**]_0_ = 0.11 M

Rate constants are:

*k*_2_ = 5.0E+09 M^-1^ s^-1^

*k*_3_ = 2.0E+06 M^-1^ s^-1^

*k*_4_ = 2.0E+06 M^-1^ s^-1^

*k*_5_ = 1.0E+09 M^-1^ s^-1^

*k*_6_ = 2.0E+06 M^-1^ s^-1^

*k*_7a_ = 1.0E+03 M^-1^ s^-1^

*k*_7b_ = 2.0E+06 M^-1^ s^-1^

*k*_8_ = 5.0E+09 M^-1^ s^-1^

*k*_9_ = 1.0E+00 M^-1^ s^-1^

*k*_10_ = 1.0E+00 M^-1^ s^-1^

For the addition of cyclohexyl radical (**9**) to acrylonitrile we select *k*_3_ = 2.0E+06 M^-1^ s^-1^ as this appears to be the most appropriate value according to Simulation III. The results of the simulations are collected in Tables S2a and S2b.

**Table S2a**. Impact of different initiation rate constants *k*_1_ on the results of the Giese reaction shown in Scheme S1.

| *k_1_*  (s^-1^) | [**9**](1s)  (M) | [**11**](1s)  (M) | [**13**](1s)  (M) | [**9**](50%)  (M) | [**11**](50%)  (M) | [**13**](50%)  (M) |
| --- | --- | --- | --- | --- | --- | --- |
| 1.0E-08 | 1.761E-11 | 1.832E-13 | 4.351E-10 | 1.362E-11 | 2.568E-13 | 3.073E-10 |
| **2.0E-08** | **2.490E-11** | **2.590E-13** | **6.155E-10** | **1.918E-11** | **3.615E-13** | **4.346E-10** |
| 3.0E-08 | 3.049E-11 | 3.172E-13 | 7.538E-10 | 2.342E-11 | 4.413E-13 | 5.323E-10 |
| 4.0E-08 | 3.520E-11 | 3.662E-13 | 8.704E-10 | 2.697E-11 | 5.081E-13 | 6.146E-10 |
| 5.0E-08 | 3.935E-11 | 4.093E-13 | 9.731E-10 | 3.009E-11 | 5.667E-13 | 6.872E-10 |
| 6.0E-08 | 4.310E-11 | 4.483E-13 | 1.066E-09 | 3.289E-11 | 6.195E-13 | 7.528E-10 |
| 7.0E-08 | 4.655E-11 | 4.842E-13 | 1.151E-09 | 3.545E-11 | 6.677E-13 | 8.132E-10 |
| 8.0E-08 | 4.975E-11 | 5.175E-13 | 1.231E-09 | 3.783E-11 | 7.124E-13 | 8.694E-10 |
| 9.0E-08 | 5.276E-11 | 5.488E-13 | 1.305E-09 | 4.006E-11 | 7.543E-13 | 9.221E-10 |
| 1.0E-07 | 5.561E-11 | 5.785E-13 | 1.376E-09 | 4.216E-11 | 7.938E-13 | 9.719E-10 |

**Table S2b**. Impact of different initiation rate constants *k*_1_ on the results of the Giese reaction shown in Scheme S1.

| *k_1_*  (s^-1^) | CL(50%) | *t_1/2_*  (s) | [**3**]  (M) | [**8**]  (M) | [**3**]/[**8**] | yield **3**  (%)^[a]^ |
| --- | --- | --- | --- | --- | --- | --- |
| 1.0E-08 | 26029 | 3224 | 0.0938 | 0.00417 | 22.49 | 93.8 |
| **2.0E-08** | **18425** | **2288** | **0.0938** | **0.00415** | **22.60** | **93.8** |
| 3.0E-08 | 15028 | 1874 | 0.0938 | 0.00413 | 22.71 | 93.8 |
| 4.0E-08 | 13014 | 1627 | 0.0938 | 0.00412 | 22.77 | 93.8 |
| 5.0E-08 | 11642 | 1458 | 0.0938 | 0.00411 | 22.82 | 93.8 |
| 6.0E-08 | 10626 | 1334 | 0.0938 | 0.00410 | 22.88 | 93.8 |
| 7.0E-08 | 9838 | 1237 | 0.0938 | 0.00410 | 22.88 | 93.8 |
| 8.0E-08 | 9203 | 1159 | 0.0938 | 0.00409 | 22.93 | 93.8 |
| 9.0E-08 | 8675 | 1094 | 0.0938 | 0.00408 | 22.99 | 93.8 |
| 1.0E-07 | 8232 | 1040 | 0.0938 | 0.00408 | 22.99 | 93.8 |

[a] calculated relative to [**1**]_0_ = 0.1 M.

Perusal of the radical concentrations collected in Table S2a clearly shows that the concentrations of the chain carrying radicals **9**, **11**, and **13** increase systematically with increasing initiation rate constant *k*_1_. This is true for the radical concentrations at the beginning of the reaction at *t* = 1s, and also at the reaction half life *t*_1/2_, where 50% of iodide **1** have been consumed. That the reaction half life *t*_1/2_ decreases systematically with increasing initiation rate constant *k*_1_ reflects the general increase in the reaction rates of the chain process with higher concentrations of chain-carrying radicals. In quantitative terms, the increase in *k*_1_ by a factor of 10 reduces *t*_1/2_, by a factor of 3.1. Despite these quite significant changes it should be emphasized that the three chain-carrying steps responsible for product formation still "tick" at approximately the same rate. For the last entry in Table S2a (that is for *k*_1_ = 1.0E-07 s^-1^), for example, we find the following reaction rates at the 50% turnover point:

r(3) = *k*_3_[**9**][**2**] = 2.0E+06 M^-1^ s^-1^ x 4.216E-11 M x 0.451 M = 3.803E-05 mol s^-1^

r(4) = *k*_4_[**13**][**4**] = 2.0E+06 M^-1^ s^-1^ x 9.719E-10 M x 0.019 M = 3.690E-05 mol s^-1^

r(5) = *k*_5_[**11**][**1**] = 1.0E+09 M^-1^ s^-1^ x 7.938E-13 M x 0.05 M = 3.970E-05 mol s^-1^

The small differences between the three reaction steps are, as mentioned before, due to the influence of the side reaction chains intertwined with the main (product forming) chain process. Higher reaction rates due to higher radical concentrations are also true for the two side reactions considered here. Both side reactions show a first-order dependence on the respective radical concentrations, as do the reactions steps of the main chain. An increase in radical concentrations thus has the same accelerating affect on both, which implies that the product distribution (and thus the yield of product **3**) shows very little influence on the initiation rate constant. The reaction therefore remains highly selective for the formation of addition product **3**, which attests to the robust nature of the overall transformation.

A somewhat surprising result in Table S2b is the reduction of the chain length as measured by the rate of product forming reaction step r(4) and the most prominent chain breaking radical recombination step r(8f), which involves the dimerization of radical **13**. The chain length determined at 50% turnover reduces from CL(50%) = 26029 for the smallest initiation rate constant of *k*_1_ = 1.0E-08 s^-1^ to CL(50%) = 8232 for the largest initiation rate constant of *k*_1_ = 1.0E-07 s^-1^. This change of 26029/8232 = 3.2 is due to the fact that the increased radical concentration of adduct radical **13** (by a factor of 9.719E-10/3.073E-10 = 3.2) enters in first order in chain reaction step r(4), but in second order in the termination step r(8f).

**Conclusions**

The microkinetics simulations presented here document in detail that the Giese reaction shown in Scheme S1 shows all characteristic of a "typical" radical chain process. The strongest control element in this transformation is the requirement that all steps of an established radical chain process have to proceed at (almost) identical rates in order to propagate productively. This is true even in the presence of additional chain processes (such as those of competing side reaction) that compete for the same chain-carrying radicals as the main chain process.

The concentrations of chain-carrying radicals vary quite significantly and respond to (a) the rate of initiation (which commonly decreases with increasing substrate turnover); (b) the concentrations of reactants (which also decrease with increasing substrate turnover); and (c) the requirement of identical rates for all chain steps. The last of these requirements has the largest impact on radical concentrations. The best value for the rate constant for addition of cyclohexyl radical (**9**) to acrylonitrile (**2**) appears to be *k*_3_ = 2.0E+06 M^-1^ s^-1^ when comparing predicted and experimentally found product yields. Higher initiation rates lead to higher concentrations of chain-carrying radicals and, thus, faster product formation, but have practically no impact on product yields (or more correctly: the ratio of addition product to reduced iodide).

**Technical details**

All microkinetics simulations have been performed with COPASI 4.44 (build 298).^[11]^ For time course simulations with reaction times up to *t* = 3 s an interval size of Δ*t* = 0.01 s was used, while all longer simulations use an interval size of Δ*t* = 0.25 s.

**References**

[1] B. Giese, J. A. Gonzalez-Gomez, T. Witzel, *Angew. Chem. Int. Ed.* **1984**, *23*, 69 - 70.

[2] B. Giese, *Angew. Chem. Int. Ed.* **1985**, *24*, 533 - 565.

[3] C. Chatgilialogly, K. U. Ingold, J. C. Scaiano, *J. Am. Chem. Soc.* **1981**, *103*, 7739 - 7742.

[4] L. J. Johnston, J. Lusztyk, D. D. M. Wayner, A. N. Abeywickreyma, A. L. J. Beckwith, J. C. Scaiano, K. U. Ingold, *J. Am. Chem. Soc.* **1985**, *107*, 4594 - 4596.

[5] M. Newcomb, *Tetrahedron* **1993**, *49*, 1151 - 1176.

[6] A. Salikhov, H. Fischer, *Appl. Magn. Reson.* **1933**, *5*, 445 - 455.

[7] K. U. Ingold, J. Lusztyk, J. C. Scaiano, *J. Am. Chem. Soc.* **1984**, *106*, 343 - 348.

[8] F. Julia, T. Constantin, D. Leonori, *Chem. Rev.* **2022**, *122*, 2292 - 2352.

[9] T. Caronna, A. Citterio, M. Ghirardini, F. Minisci, *Tetrahedron* **1977**, *33*, 793 - 796.

[10] B. Giese, *Angew. Chem. Int. Ed.* **1983**, *22*, 753 - 764.

[11] COPASI 4.44 (Build 295). COPASI is a simulator for biochemical networks accessible at https://copasi.org/. It is a joint project by the Hoops group (Biocomplexity Institute of Virginia Tech), the Mendes group (UCONN School of Medicine), the Kummer, and Sahle groups (University of Heidelberg).

[12] B. Giese, G. Kretzschmar, *Chem. Ber.* **1984**, *117*, 3160 - 3164.

[13] E. S. N. Cotter, N. J. Booth, C. E. Canosa-Mas, D. J. Gray, D. E. Shallcross, R. P. Wayne, *Phys. Chem. Chem. Phys.* **2001**, *3*, 402 - 408.
